# Synergistic Epistasis and Systems Biology Approaches to Uncover a Pharmacogenomic Map Linked to Pain, Anti-Inflammatory and Immunomodulating Agents (PAIma) in a Healthy Cohort

**DOI:** 10.1007/s10571-024-01504-2

**Published:** 2024-11-06

**Authors:** Alireza Sharafshah, Majid Motovali-Bashi, Parvaneh Keshavarz, Kenneth Blum

**Affiliations:** 1 Division of Genetics, Department of Cell and Molecular Biology and Microbiology, Faculty of Biological Science and Technology , University of Isfahan, Isfahan, Iran; 2 Cellular and Molecular Research Center, School of Medicine, Guilan University of Medical Sciences, Rasht, Iran; 3 Division of Addiction Research and Education, Center for Sports, Exercise, and Mental Health, Western University Health Sciences, Pomona, CA USA; 4 Department of Psychiatry, Wright State University Boonshoft School of Medicine, Dayton, OH USA

**Keywords:** Pharmacogenomics, Gene–gene interactions, Variant, MDR, WES

## Abstract

The global public health addiction crisis has been stark, with over 932,400 deaths in the USA and Canada from opioid overdose since 1999–2020, surpassing the mortality rates at the top of the HIV/AIDS epidemic. Both nations exhibit opioid consumption rates significantly above the norm for developed countries. Analgesic type of opioids present both therapeutic benefits and substantial health risks, necessitating balanced drug regulation, careful prescribing, and dedicated opioid stewardship. The role of the cytochrome P450 2D6 (CYP2D6) system (Enzymatic functions) in metabolizing opioids highlights the potential of genotype-guided analgesia. By integrating Pharmacogenomics (PGx), this approach aims to optimize pain management, enhance safety, and reduce addiction risks. This understanding prompted the utilization of multifactor dimensionality reduction (MDR) to explore a range of phenotypes including PGx and gene–gene interactions (GGI) in a healthy cohort, thereby personalizing pain management strategies. The study sampled 100 unrelated healthy Western Iranians and 100 individuals from the 1000 Genome Project. Pre-testing involved searching for PGx annotations (variants associated with drug-gene-diseases) related to pain sensitivity and inflammation using the PharmGKB database, which identified 128 relevant genes. A questionnaire helped select 100 participants who had never used potent opioids but also other psychoactive agents (e.g., nicotine, amphetamines, etc.) and disease-related drugs. Whole-exome sequencing (WES) was then employed to analyze these genes in an Iranian cohort. Further analyses included MDR for identifying synergistic gene annotations and GGI for exploring complex gene interactions through the Visualization of Statistical Epistasis Networks (ViSEN). The study identified a Pain, Anti-Inflammatory, and Immunomodulating agents (PAIma) panel from the 128 genes, resulting in 55,590 annotations across 21 curated pathways. After filtering, 54 significant structural or regulatory variants were identified. This research also highlighted novel gene relationships involving the *CYP3A5* gene, hsa-miR-355-5p, Paliperidone, and *CYP2D6*, which warrant further investigation. This study offers a novel pharmacogenetic framework that could potentially transform opioid prescribing practices to mitigate misuse and enhance personalized pain management. Further validation of these findings from multi countries and ethnic groups could guide clinicians in implementing DNA-based opioid prescribing, aligning treatment more closely with individual genetic profiles.

**Graphical abstract:**

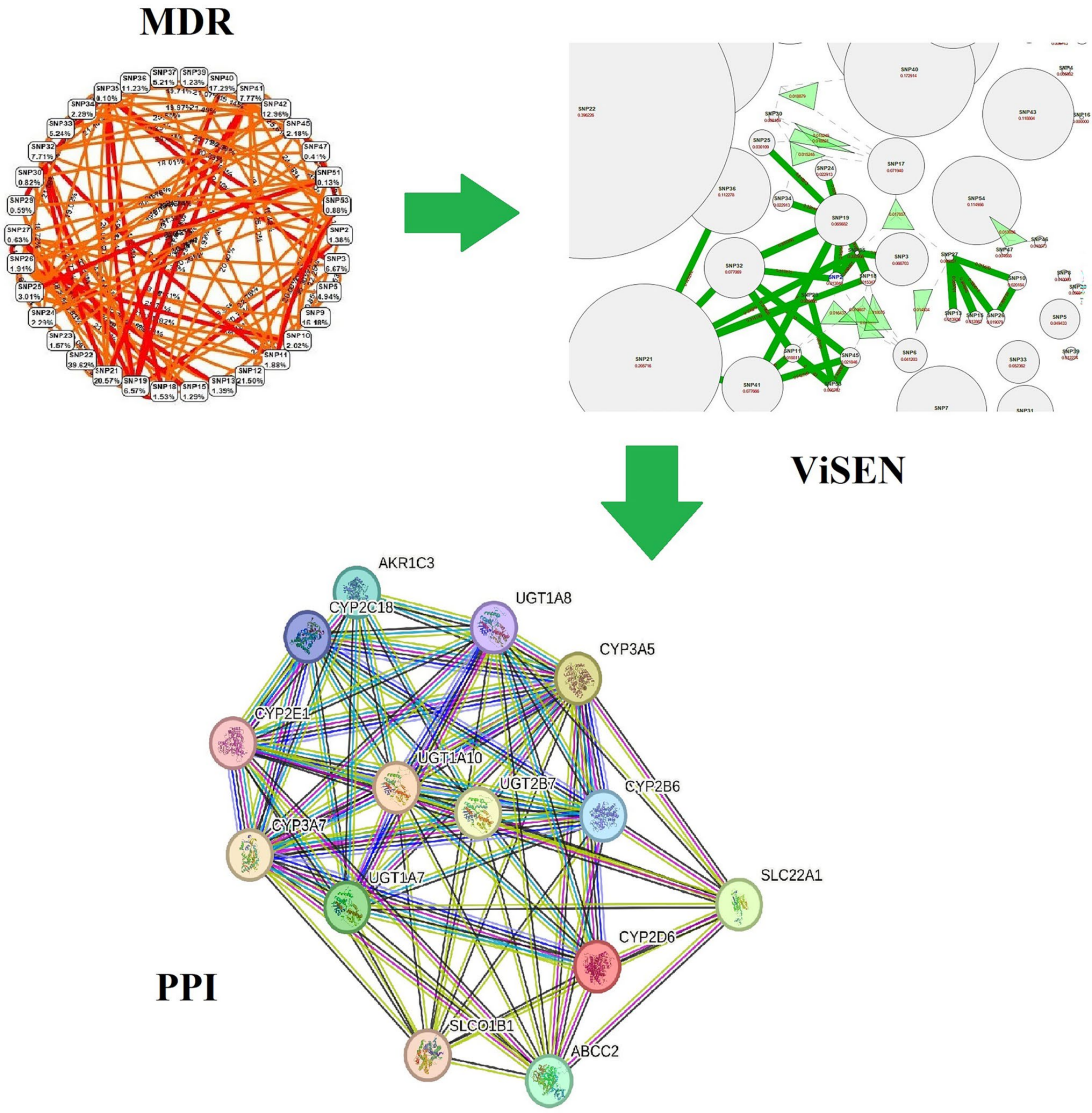

**Supplementary Information:**

The online version contains supplementary material available at 10.1007/s10571-024-01504-2.

## Introduction

Opioid misuse and abuse constitute a remarkable crisis in global public health, particularly in the United States (US). Clinicians are increasingly encouraged to focus on the treatment and prevention of opioid use disorders (OUDs). Notably, methadone, naltrexone, and buprenorphine are the three drugs which the Food and Drug Administration (FDA) has approved for the treatment of OUD (Oesterle et al. 2019). Opioid agonist therapy (OAT) has been documented as the cornerstone of OUD treatment. OAT is an approach regulating opioid receptors to lessen cravings and substance use. While OAT preserves opioid dependency, it effectively mitigates the negative consequences of substance abuse and overdose (Lee et al. 2022). Given the worst opioid epidemic in the US and other countries, also, the rising number of overdose deaths, there is an urgent need for more sustainable and non-addictive effective treatments for OUD.

It is indeed thoughtful to encourage neuromodulatory interventions to motivate the neural circuitry of addiction functioning in the dorsolateral prefrontal cortex and deeper structures of the mesolimbic system to restrain desire and decrease usage (Lee et al. 2022). Other potential therapies for OUD include targeting distinct dopamine-related addiction system components, identifying susceptible genes and altering gene products, and employing immunizations as immunotherapy to lessen the addictive effects of illicit drugs. Additional clinical evidence is required to confirm the safety and efficacy of these medications in OUD, however, these suggested innovative treatments morph opioid receptors and provide promise for a more long-lasting OUD therapy (Lee et al. 2024).

Detecting polymorphisms, or sequence variations, which increase disease risk, is among the most complicated challenges in human genetics. When it comes to uncommon Mendelian single gene diseases including cystic fibrosis (CF) or sickle-cell anemia, the association between phenotype and genotype is simply visible since the mutant-carrying genotypes directly cause the disease. Unfortunately, this type of association is exceedingly difficult to quantify in the case of prevalent, complicated diseases like hypertension, diabetes, or multiple sclerosis. This is because the disease seems to be the outcome of several genetic factors and social-economic and -spiritual environmental variables. Indeed, gene–gene interaction, or epistasis, is increasingly documented to be essential in the genetic framework of prevalent disease and disorders (Moore 2003; Sing et al. 2004; Thornton-Wells et al. 2004). This challengeable issue also exists in pharmacogenomics studies that could lead to a personalized approach to for example pain management (Consortium 2004; Wilke et al. 2005c).

In the framework of human evolution, prescription drug usage represents a moderately modern phenomenon, offering a substantial item for recently identifiable interactions between adverse environmental conditions (novel medications; epigenetics) and extremely polymorphic genotypes (archaic genes). Polymorphisms in drug-metabolizing enzymes (DMEs) may rank amongst the most common inherited risk factors of disease development if adverse drug reactions (ADRs) and treatment defects are recognized as distinct disorders (Wilke et al. 2005b). Genetic variants with functional roles are not limited to catabolism or metabolism. Genetic diversity affects receptor affinity and a number of intricate processes related to drug disposition, including Absorption, Distribution, Metabolism, and Excretion (ADME). Moreover, genetic variations in known or even unknown pharmacodynamics pathways (molecular signal transduction) may provide variances in outcomes that are therapeutically discernible. Even in the context of medications having clinically mild ADR and somewhat broad therapeutic indicators, sophisticated computer algorithms may reveal hitherto undetected gene–gene interactions resulting in the phenotypic change by considering these complex additional layers. (Wilke et al. 2005c).

In datasets containing categorical independent variables, such as single nucleotide polymorphisms (SNPs) along with other sequence variations, such as insertions, deletions, and so forth, in addition to environmental data that can be represented as categorical variables, Multifactor Dimensionality Reduction (MDR) was established for detecting gene–gene or gene-environment connections. When studying disorders that impact humans, the dependent variable, or endpoint, needs to be binary, meaning it may be either both cases or controls. When evaluating pharmacogenomics data, MDR can be applied with "response/non-response" or "toxicity/no toxicity" metrics. MDR may also be used for any dataset that serves two distinct therapeutic objectives (Motsinger and Ritchie 2006).

In the current investigation, MDR and its multiple modifications have been employed to investigate an extensive spectrum of phenotypes, including pharmacogenetics (Wilke et al. 2005c; Dai et al. 2013). As healthcare professionals and lawmakers work harder to improve drug safety, scientists and researchers are being more motivated to enhance the administration and analysis of population-based toxicogenomic datasets (Lord and Papoian 2004). Furthermore, high-throughput genotyping efficacy and big cohort investigations are improving as science approaches better tools towards uncovering gene -gene interactions (genotyping) in a very positive manner to help explore in deep silico analyses (McCarty et al. 2005). As these factors interact, analytical omics linked informatics face a growing demand for high-quality computational tools that have the potential to help the finding of formerly unknown gene–gene interactions in the framework of drug toxicities (Wilke et al. 2005a). In the future,using MDR and similar methods, it could be possible to create better gene-based dosage models and promote safer medication prescription practices through the employment of individual drug sensitivity profiles. (Hoh et al. 2001; Culverhouse et al. 2004; Wilke et al. 2005c).

Thus, our deep in silico investigation aimed to broaden the scope by utilizing MDR in a pharmacogenomics-based gene–gene interaction. This has been accomplished by designing a signaling pathway panel (PAIma) on the Whole-Exome Sequencing (WES) results of a randomized healthy non-psychoactive abusing (nicotine, amphetamines, antidepressants, etc.) Western Iranians that have been specifically identified for never imbibing powerful opioid analgesic medications enabling a novel stratified population. Thus, we embarked on an exploratory PGx map in this cohort to putatively identify genotypic risk and therapeutic targets to attenuate opioid misuse.

## Material and Methods

### Sampling and Data Collection

The participants of the current study included 100 healthy individuals who provided their DNA samples to the medical genetic laboratory in Kermanshah Province; thus, all of the samples were Kurdish. During a one-year screening, we recruited healthy individuals who were referred to the laboratory for a WES test.

A printed informed consent was completed from every individual. This research gained the ethical approval of University of Isfahan and Biomedical Research Ethics Committee [IR.UI.REC.1402.092]. The primary subjects were 150 people without any significant manifestations for a specific disease and their blood tests were normal for routine biochemical factors. Based on the included variant annotations and following adjusting for each related drug with its own PGx variant, 47 unique drugs and 2 drug families (antiandrogens and antiepileptics) were obtained. To determine ever using an opioid, a questionnaire was designed to determine the background of each subject. Specifically, we were able to identify an individual’s past history related to ever taking any addictive drugs (methadone, morphine, methamphetamine, nicotine, etc.) and or lifetime substance use disorder. Following the completion of this questionnaire, 50 individuals were discarded/excluded for further investigation due to their use of Nicotine (Cigarette smoking), celecoxib, rofecoxib, clopidogrel, and lorazpam and addiction history. Accordingly, all of the questionnaires were completed by subjects and checked by the laboratory staff and was further inspected by a staff Pharmacist (Table 1).

**Table 1:** Questionnaire for exclusion of potential subjects related to usage of listed drugs (both addictive and no addictive pharmaceuticals)

Category	Drug	Usual consumption	Occasional consumption
Anti-Allergy	Dextromethorphan	0	0
Anti-Allergy- NSAID	Celecoxib	15	20
Anti-Allergy- NSAID	desmethylnaproxen	0	2
Anti-Allergy- NSAID	Rofecoxib	0	2
Antiviral medicine- hepatitis B	Sofosbuvir	0	0
Antiviral medicine-HIV	Atazanavir	0	0
Antiviral medicine-HIV	Ritonavir	0	0
Antiviral medicine-HIV	Efavirenz	0	0
Antiviral-treat hepatitis B virus and HIV	Tenofovir	0	0
Blood	Telmisartan	0	0
Blood- atherosclerosis	Fluvastatin	0	0
Blood- Treat for thalassemia, sickle-cell disease, or anemia	Deferiprone	0	0
Blood- treat high blood pressure	Hydrochlorothiazide	0	0
Blood-an antiplatelet medicine-Acute coronary syndrome	Clopidogrel	0	1
Blood-Diabetes	Metformin	0	0
Blood-statin	Pitavastatin	0	0
Blood-statin	Rosuvastatin	0	0
Blood-treating iron toxicity	Deferasirox	0	0
Chemoterapeutic-treat colorectal cancer	Oxaliplatin	0	0
Chemotherapeutic	Idarubicin	0	0
Chemotherapeutic	Irinotecan	0	0
Chemotherapeutic agent	Capecitabine	0	0
Chemotherapeutic- anti metabolites	Fludarabine	0	0
Chemotherapeutic	Doxorubicin	0	0
Chemotherapeutic	Carboplatin	0	0
Chemotherapeutic	Gemcitabine	0	0
Chemotherapeutic	Imatinib	0	0
Chemotherapeutic-anti metabolites	Fluorouracil	0	0
Chemotherapeutic-antineoplastic anti-metabolite	Cytarabine	0	0
Chemotherapeutic treatment of AML	Ozogamicin	0	0
Chenoterapeutic-AML	Gemtuzumab	0	0
Cheomotherpeutic	Cyclophosphamide	0	0
Heart Diseases	Carvedilol	0	0
Heart Diseases	Bufuralol	0	0
Hormone- menopausal hormone therapy	17beta-estradiol glucuronide	0	0
Opioid	Nicotine	10	21
Opioid	Methadone	0	0
Opioid	Methamphetamine	0	0
Opioid	Morphine	0	0
Psychiatric- schizophrenia/ Major Depressive disorder	Aripiprazole	0	0
Psychiatric- Anti-anxiety	Lorazepam	0	4
Psychiatric-Antipsychotic	Clozapine	0	0
Psychiatric-Anti-psychotic	Valproic acid	0	0
Psychiatric-anti-seizure/anti-epilepsy drug	Lamotrigine	0	0
Transplantation- stop graft rejection in stem cell transplantation and to treat a variety of malignancies	Thiotepa	0	0
Transplantation- anti rejection	Tacrolimus	0	0
Transplantation- anti rejection	Cyclosporine	0	0
Transplantation- anti rejection	Mycophenolate mofetil	0	0

The total population that completed the questionnaire was 150 people that provided their DNA samples for further testing. The subjects were also asked if they were addicted to alcohol or any other substance in their lifetime. Each drug status was checked with a pharmacist based on the subjects’ responses. NSAID refers to Nonsteroidal anti-inflammatory drug and AML stands for Acute Myeloid Leukemia. Usual consumption and occasional consumption indicate the number of individual(s)

### Exclusion/Inclusion Criteria

#### Inclusion

The following were the inclusion requirements for the individual: (1) the subject had to be more than 20 years old, (2) have normal blood test results (based on routine biochemical indexes like CBC, WBC, RBC, FBS, HbA1c, CRP, etc.), lack of having any disease-related phenotypes (major genetic manifestations that have been diagnosed by a healthcare specialist such as common syndromes, metabolic, and musculoskeletal disorders), (3) not have any consanguinity status in their parents’ marriages and (4) life-time non-usage of psychoactive drugs (see Table 1),

#### Exclusion

Exclusion criteria requirements included: (1) healthy participants with patient children who might be a heterozygous carrier of a pathogenic mutation related to an autosomal recessive disease; (2) subjects with a consanguineous pedigree in terms of parent–child relationship; and (3) individuals under the age of 20. The fundamental data for this research was obtained from PharmGKB, which consisted of curated pathways classified to reflect agents (PAIma) linked to pain, anti-inflammatory, and immunomodulation.

It is indeed noteworthy that along with the pathways for anticancer, neurological, and cardiovascular drugs by 2023, this category, which has 37 signaling pathways (21 curated pathways), is one of the most promising evidence-based pathways. PharmGKB pathways are evidence-based diagrams that depict a drug’s pharmacokinetics (PK) “and/or” pharmacodynamics (PD) concerning significant (or possibly significant) Pharmacogenomics (PGx) associations.

### WES Tests and NGS Analyzing Strategies

A WES test was performed on every individual to identify pathogenic variants: a filter-based approach to extract and purify genomic deoxyribonucleic acid (gDNA) from the blood samples of subjects, which was then analyzed. One gram (1.0 g) of gDNA was employed for the preparation of DNA. Moreover, to create sequencing datasets, the Agilent SureSelect- Human All ExonV7 Kit (Agilent Technologies, USA) was employed, and subsequently the sample attribute sequences and the x-index codes were connected. The DNAs were fragmented into 180–280 bp segments utilizing hydrodynamic shear method (Covaris, USA). Exonuclease/polymerase processes reduced residual overhangs, and enzymes were eliminated from the nest. Adapter oligonucleotides were successfully ligated following the adenylation of the DNA fragments’ 3ʹ ends. Additionally, in a PCR procedure, DNA fragments with adaptor molecules attached at each end were deliberately chosen. In order to prepare the collected libraries for hybridization, index tags were added by a PCR amplification. The products were measured using the Agilent 2100 Bioanalyzer and Agilent High Sensitivity DNA Assay after they were purified using the Beckman Coulter AMPure XP system. Of note, the verified libraries were put onto the Illumina NovaSeq 6000 sequencer. Next, using an HP server (a Generation G9 with a Unix-based operator system), data quality control, analysis, and interpretation were executed. With Ubuntu (ver. 22.04.2), NGS analysis was performed on each fastq file using filtered-based command-line procedures including the genomic packages fastqc, IlluQC, Cutadapt, Alignment, Post-Alignment, BQSR, Variant-calling, VQSR, Annotation with ANNOVAR, and Filtering. Three levels of annotation were used including region-level (cytoBand), gene-level (refGene), and frequency-level (cytoBand, exac03, dbnsfp30a, avsnp150, clinvar_20221231, regsnpintron, and icgc28) databases (Wang et al. 2010). Following the combining of the 128 PAIma gene candidate panel on the Variant Call Format (VCF) files, the Reference Sequence (RS) IDs of the variants were used to apply commands, and the genotypes for each individual’s variant were retrieved.

### In Silico and Statistical Analyses

The in silico investigations were performed on the 128 candidate PAIma genes to uncover the novel interactions at different levels that included Gene–Gene Interactions (GGIs) by MDR (version 3.0.2), signaling pathways by Cytoscape (version 3.10.1), Protein–Protein Interactions (PPIs) via STRING-MODEL (version 12), Gene-miRNA Interactions (GMIs) through miRTargetLink2 (version 2.0), and finally, Protein-Drug Interactions (PDIs) and Protein-Chemical Interactions (PCIs) both via Network Analyst (version 3) (Zhou et al. 2019). Prior to performing MDR analysis, we employed a PS power and sample size (version 3.1.6) (available at: https://biostat.app.vumc.org/wiki/Main/PowerSampleSize#Windows) to calculate the power of study including all the refined variants based on the following design: Dichotomous, independent, case–control, and Fischer’s Exact Test with the statistical indicators including α (Type I error probability), *p*_0_ (MAF), n (number of cases), m (ratio of control to case patients), and Ψ (OR) were performed.

Epistasis and synergism were calculated via MDR analysis through the MDR software (version 3.0.2) (Institute for Quantitative Biomedical Sciences, USA) (Ritchie et al. 2003). To prepare the appropriate file for MDR, the genotype frequencies obtained from our case group population and the all phase III individuals of the 1000 genome project were considered as the control group. To develop the ratio of 1:1 for this case–control analysis, genotype frequencies of the control group were adjusted for 100 individuals. While the case group had not any phenotypic disease; we were cognizant of the pharmacogenetic diversity of this specific Iranian population compared to known population pharmacogenomics status. The optimal model for predicting susceptibility to the PAIma gene list was selected according to the lowest grouping error in a training set (known as vector R, the element of which indicates samples in the data sets), and a 10-folded cross-validation was considered to assess prediction accuracy. The alpha level set for our analyses was at the 95% level (*p* < 0.05).

Concerning the ratio of cases/controls, the genotypic combination was categorized as either low or high risk to help identify the risk evaluation. Finally, a dendrogram, Fruchterman-Reingold plot and circle graph were generated for illustrating the chosen model according to an information theory (Moore et al. 2006). Every connection among variants indicates the entropy risk as percentage of the overall dataset. Positive percentage rates were thought to have a synergistic connection, while scores of 0i or less were seen to be antagonistic or redundant. The fact that the paired percent rate is larger than the individual rate, as shown by the more accurate model, is noticeable (Hahn et al. 2003).

In summary, entropy measurements are used to quantify the volume of information regarding the case–control status defined by unique characteristics. Importantly, it is worthwhile to mention that a negative rate denotes (correlation redundancy owing to linkage disequilibrium: LD), whereas a positive data gain denotes a significant synergistic or non-additive impact (epistasis for instance). Notably, as indicated in the result section the red line linking the two distinct SNPs suggests a highly synergistic interplay (Hu et al. 2011, 2013a). By integrating Pharmacogenomics (PGx), this approach aimed to optimize pain management, enhance safety, and reduce addiction risks. This understanding prompted the utilization of multifactor dimensionality reduction (MDR) to explore a range of phenotypes including PGx and gene–gene interactions (GGI) in a healthy cohort, thereby personalizing pain management strategies.

## Results

### Data Mining and Analyzing the WES Results

Data mining of 21 curated PAIma pathways from the PharmGKB database (https://www.pharmgkb.org/) revealed 55,590 annotations, 900 significant variants affecting FDA-approved drugs, and 128 genes. After several filtrations, 128 genes were retained as the main gene list for the WES test analysis. The PAIma panel of genes include: *ABCB1, ABCC2, ABCC3, ABCC4, ABCG2, AKR1B1, AKR1C3, AMACR, ATF2, ATF3, BATF, CES1, CES2, CNR1, CNR2, CYP1A2, CYP1A1, CYP2A6, CYP2C18, CYP2B6, CYP2C19, CYP2D6, CYP2C9, CYP2C8, CYP2E1, CYP3A, CYP3A4, CYP3A7, CYP3A5, FAAH, FKBP1A, FOS, FOSB, FOSL1, FOSL2, GSTA1, GSTM1, GSTP1, GSTT1, HPGDS, IL2, IMPDH1, IMPDH2, JUN, JUNB, JDP2, JUND, MAFB, MAFA, MAFG, MAFF, MAFK, MAF, MAP2K4, MAP2K3, MAP2K6, MAP3K1, MAP2K7,MAP3K7, MAPK8, MAP3K11, MAPK14, NFATC2, NFATC1, NFATC4, NFKB2, NFKB1, NOS1, NOS2, NOS3, NRL, PLA2G2A, PLA2G4A, PPP3CA, PPIA, PPP3CC, PPP3CB, PPP3R1, PTGDR, PPP3R2, PTGDR2, PTGER1, PTGDS, PTGER2, PTGER4, PTGER3, PTGES, PTGES3, PTGES2, PTGIR, PTGFR, PTGIS, PTGS2, PTGS1, REL, RELB, RELA, S1PR1, S1PR3, S1PR5, SLC22A1, SLC22A11, SLC22A6, SLC22A7, SLC22A8, SLC22A9, SLCO1B1, SLCO1B3, SLCO2B1, SULT1A1, SULT1A3, SULT1A4, SULT1E1, SULT2A1, TBXA2R, TBXAS1, TGFB1, UGT1A10, UGT1A1, UGT1A3, UGT1A7, UGT1A6, UGT1A8, UGT2B15, UGT1A9, UGT2B17, UGT2B7* and *UGT2B4,*.

By excluding non-coding and synonymous variants, 54 candidate variants were identified that differed from the reference genome (hg38) based on varying Minor Allele Frequency (MAF) estimates for all 100 WES results. Based on the variant functions, there were just 48 nsSNVs out of 54. Besides, 6 variants were either a splicing (rs2270860, rs776746, and rs4513095) or highly structure-altering missense mutations changing amino acids affecting the final protein product, including 2 stop-gained [a mutation that cause a premature termination codon] (rs17863778 and rs145014075) and 1 frameshift [a mutation that induces an insertion/deletion resulting in changing the triplet reading codons] (rs11572078) variant. Moreover, it was found that some nsSNVs had overlapping functions either functional (missense) or regulatory [promoter, transcription binding site, enhancer, and CCCTC-binding factor (CTCF)]. As mentioned earlier, rs17863778 (*UGT1A7*) and rs145014075 (*CYP2A6*) are stop-gained variants, rs11572078 (*CYP2C8*) is a frameshift, and rs2270860 (*SLC22A7*), rs776746 (*CYP3A*; *CYP3A5*), and rs4513095 (*CES1*) are annotated as splicing variants (Table 2).

**Table 2:** Genes and their related variants included in PAIma panel

Variant	Gene(s)	Function	MAF	maf	Gt	Drugs
rs72551330	*UGT1A9*	nsSNV	0.009	1	T > A	Mycophenolate mofetil
rs1801030	*SULT1A1; SULT1A2*	nsSNV	0.007	0.995	C > T	
rs1751034	*ABCC4*	nsSNV	0.19	0.87	C > T	Tenofovir
rs683369	*SLC22A1*	nsSNV	0.21	0.86	G > C	Imatinib
rs4149117	*SLCO1B3*	nsSNV	0.16	0.86	T > G	Mycophenolate mofetil
rs2257401	*CYP3A7*	nsSNV	0.13	0.86	C > G	Tacrolimus
rs2515641	*CYP2E1*	nsSNV	0.14	0.8	T > C	Cytarabine; fludarabine; gemtuzumab ozogamicin; idarubicin
rs1799983	*NOS3*	nsSNV	0.31	0.73	T > C	Hydrochlorothiazide
rs628031	*SLC22A1*	nsSNV	0.39	0.66	A > G	Metformin
rs16947	*CYP2D6*	nsSNV	0.32	0.6	A > G	
rs17868323	*UGT1A10; UGT1A6; UGT1A7; UGT1A8; UGT1A9*	nsSNV	0.4	0.58	T > G	Irinotecan
rs12529	*AKR1C3*	nsSNV	0.43	0.56	C > G	Antiandrogens
rs7439366	*UGT2B7*	nsSNV	0.49	0.53	T > C	Lamotrigine
rs4292394	*UGT2B7*	nsSNV	0.49	0.52	C > G	Methadone
rs7438284	*UGT2B7*	nsSNV	0.49	0.52	A > T	Lorazepam; valproic acid
rs1135840	*CYP2D6*	nsSNV	0.43	0.49	G > C	Aripiprazole
rs6759892	*UGT1A6*	nsSNV	0.4	0.42	T > G	Deferiprone
rs1105879	*UGT1A6;UGT1A10; UGT1A8; UGT1A9; UGT1A7;*	nsSNV	0.35	0.4	A > C	Valproic acid
rs11692021	*UGT1A7;UGT1A10; UGT1A6; UGT1A9;UGT1A8*	nsSNV	0.35	0.38	T > C	Irinotecan
rs2306283	*SLCO1B1*	nsSNV	0.43	0.37	A > G	Pitavastatin
rs3740066	*ABCC2*	nsSNV	0.33	0.36	C > T	Antiepileptics
rs4244285	*UGT1A8*	nsSNV	0.21	0.33	C > G	Cyclosporine; mycophenolate mofetil
rs2070959	*UGT1A10; UGT1A7; UGT1A6; UGT1A9; UGT1A8*	nsSNV	0.32	0.31	A > G	Valproic acid
rs3745274	*CYP2B6*	nsSNV	0.26	0.29	G > T	Efavirenz
rs2273697	*ABCC2*	nsSNV	0.19	0.29	G > A	Deferasirox
rs2279343	*CYP2B6*	nsSNV	0.25	0.27	A > G	Efavirenz
rs28365063	*UGT2B7*	nsSNV	0.17	0.23	A > G	Carvedilol
rs1695	*GSTP1*	nsSNV	0.33	0.21	C > T	Fluorouracil; irinotecan; oxaliplatin
rs324420	*FAAH*	nsSNV	0.21	0.19	C > A	Methamphetamine
rs60140950	*SLCO1B3*	nsSNV	0.13	0.19	G > C	Telmisartan
rs1042028	*SULT1A1*	nsSNV	0.32	0.185	C > G	
rs1126545	*CYP2C18*	nsSNV	0.15	0.18	C > T	Clozapine
rs4244285	*CYP2C19*	nsSNV	0.15	0.16	G > A	Cyclophosphamide
rs717620	*ABCC2*	nsSNV	0.19	0.13	C > T	Antiepileptics
rs11045819	*SLCO1B1*	nsSNV	0.15	0.11	C > A	Fluvastatin
rs12208357	*SLC22A1*	nsSNV	0.07	0.1	C > T	Morphine
rs1799853	*CYP2C9*	nsSNV	0.11	0.09	C > T	Celecoxib
rs8187710	*ABCC2*	nsSNV	0.06	0.08	G > A	Rosuvastatin
rs1042008	*SULT1A1*	nsSNV	0	0.08	G > A	Desmethylnaproxen
rs3211371	*CYP2B6*	nsSNV	0	0.08	C > T	Cyclophosphamide; doxorubicin
rs1138272	*GSTP1*	nsSNV	0.08	0.065	C > T	Thiotepa
rs2306168	*SLCO2B1*	nsSNV	0.04	0.06	C > T	Rosuvastatin
rs12248560	*CYP2C19*	nsSNV	0.22	0.04	C > T	Clopidogrel
rs3842787	*PTGS1*	nsSNV	0.07	0.03	C > T	Rofecoxib
rs11568681	*ABCC4*	nsSNV	0.018	0.02	G > T	17beta-estradiol glucuronide
rs138417770	*CYP2D6*	nsSNV	0	0.005	G > C	Bufuralol; dextromethorphan
rs17863778	*UGT1A7*	Stop-gained	0.4	0.56	C > A	Atazanavir; ritonavir
rs145014075	*CYP2A6*	Stop-gained	0.04	0.06	G > T	Nicotine
rs11572078	*CYP2C8*	Frameshift	0.17	0.31	T > TA	Carboplatin; gemcitabine
rs2270860	*SLC22A7*	Splicing	0.33	0.39	C > T	Capecitabine
rs776746	*CYP3A5*	Splicing	0.11	0.06	C > T	Tacrolimus
rs4513095	*CES1*	Splicing	0.02	0.05	C > A	Sofosbuvir

The genetic details related to the 54 refined variants selected for MDR. MAF and Gt mean Minor Allele Frequency and Genotype, respectively. Remarkably, MAF reflects minor allele frequency deposited in genomic databases (dbsnp/NCBI) and MAF derived from the current study involving WES results. Also, nsSNV refers to non-synonymous Single Nucleotide Variant (missense)

In this current investigation, we explored our case–control cohort and its potential derived data from dbsnp source (https://www.ncbi.nlm.nih.gov/snp/) and plausibility that our subsequent results indicates that the likelihood of exposure in controls (*p*_0_) is 0.22 (calculated as mean MAF based on Table 2). We will be able to reject the null hypothesis that this OR equals 1 with probability (power) 0.802 if the real odds ratio (OR) for disease in exposed participants compared to unexposed ones is 2.5. The risk of Type I error for this null hypothesis test is 0.05 (α). Therefore, with a power analysis of greater than 80, we assessed this null hypothesis using a Fisher’s exact test or a continuity-adjusted chi-squared (χ^2^) statistic.

### Gene–Gene Interactions (GGIs) with MDR

**Fig. 1:**
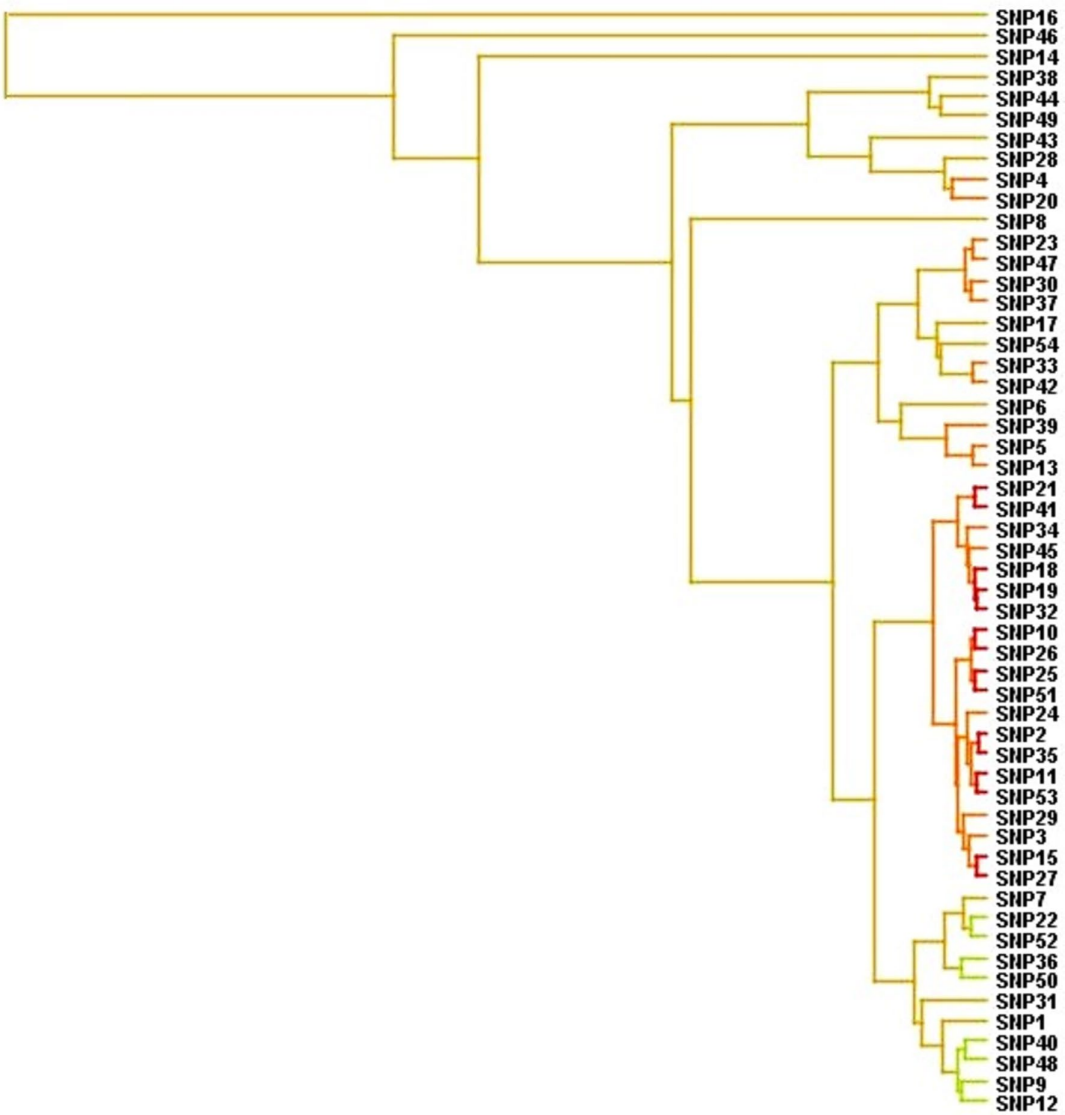
The dendrogram model of gene–gene interactions of 54 candidate variants were computed and visualized by MDR. This figure signifies the potential synergism of candidate SNPs with each other; more specifically, the red lines highlight the most significant synergic relationships. A red or orange line specifies synergistic connection, golden line characterizes additivity, and green or blue indicates redundancy. We found no blue lines

**Fig. 2:**
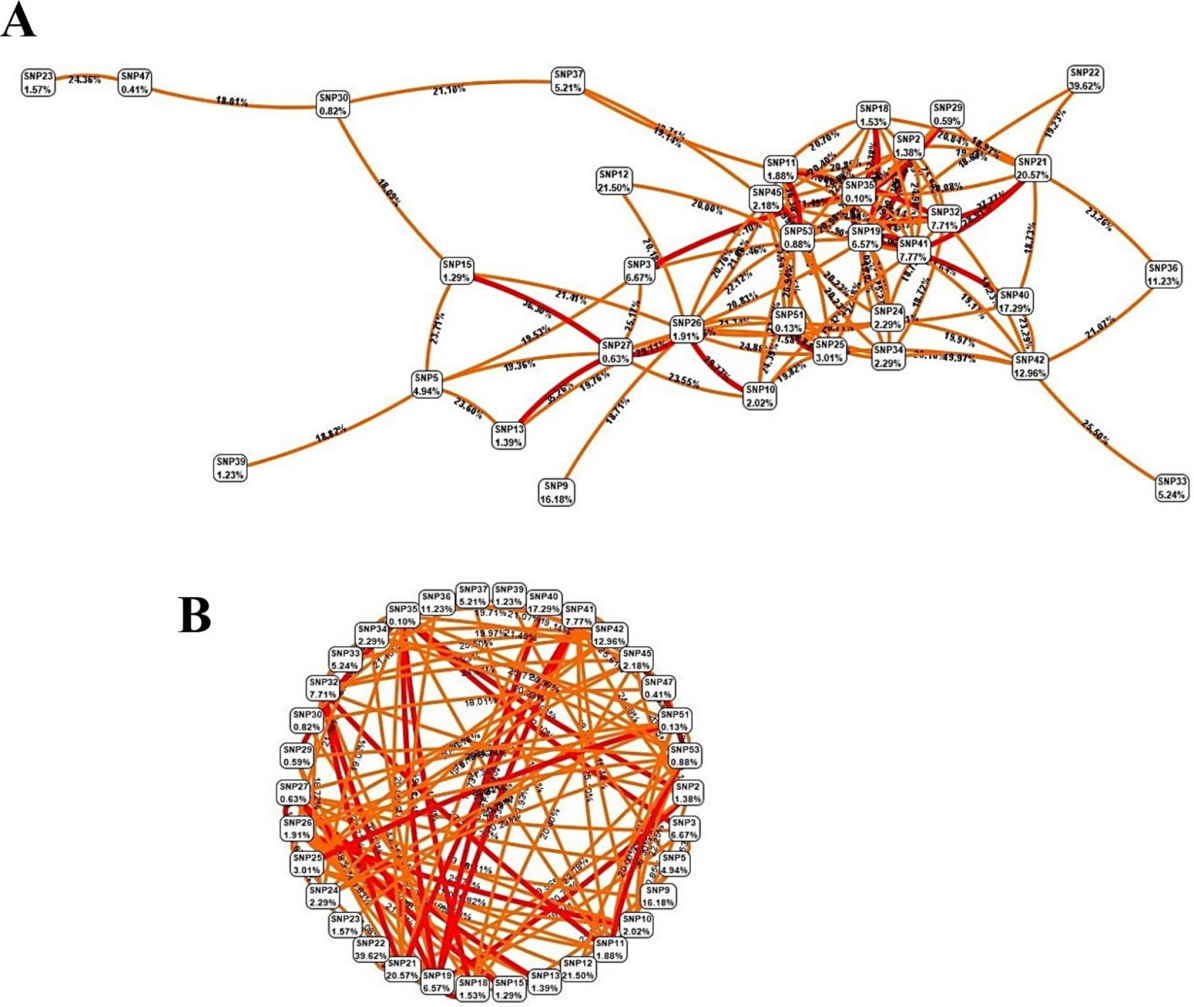
**A** Fruchterman-Reingold model of SNP-SNP synergism; **B** Circular model of SNP-SNP synergism. The high level of synergistic relationships in both models is shown with red and the order of priority is as follows: orange, gold, and green (shows reluctance). These networks signify the multiple interactions of SNPs with each other which in turn, demonstrates initial validations for precision of primary data collection

Successfully utilizing the MDR analysis enabled the Entropy-based SNP-SNP interaction network of 54 variants in a combined attribute network. The whole dataset statistics calculated by MDR were included as a balanced accuracy [the average of recall obtained on each class]: 0.99, Sensitivity: 0.98, Specificity: 1.0, Χ^2^:192.1569 (*p* < 0.0001), Precision: 1.0, Kappa: 0.98, and F-Measure: 0.9899. In a dendrogram model, the synergic relationships of the 54 final variants on each other are represented (Fig. 1). With a node visibility threshold of 0.0667 (100%), SD of 0.0787, maximum betweenness centrality of 28.32, and maximum closeness centrality of 0.76, dendrogram, Fruchterman-Reingold (Fig. 2A), and Circle (Fig. 2B) models illustrated interesting synergic relationships among some SNPs including [SNP4 (*GSTP1*_rs1138272) and SNP20 (*CYP2C9*_rs1799853)], [[SNP23 (*UGT2B7*_rs28365063) and SNP47 (*ABCC2*_rs717620)]/[SNP30 (*SLC22A7*_rs2270860) and SNP37 (*NOS3*_rs1799983)]], [SNP33 (*SLCO2B1*_rs2306168) and SNP42 (*SLCO1B3*_rs4149117)], [[SNP39 (*ABCC4*_rs1751034)] > [SNP5 (*GSTP1*_rs1695) and SNP13 (*UGT1A10*_rs1105879])]. Moreover, a synergistic cluster among 21 SNPs was found including SNP21 (*CYP3A7*_rs2257401), SNP22 (*CYP3A5*_rs776746) SNP41 (*SLCO1B1*_rs2306283), SNP34 (*UGT2B7*_rs7439366), SNP45 (*UGT1A7*_rs17863778), SNP18 (*CYP2D6*_rs1135840), SNP19 (*CYP2E1*_rs2515641), SNP32 (*SLCO1B1*_rs2306283), SNP10 (*UGT1A8*_rs2070959), SNP26 (*ABCC2*_rs3740066), SNP25 (*UGT2B7*_rs7438284), SNP51 (*SLC22A1*_rs628031), SNP24 (*UGT2B7*_rs7439366), SNP2 (*CYP2D6*-rs1135840), SNP35 (*AKR1C3*_rs12529), SNP11 (*UGT1A10*_rs17868323), SNP53 (*UGT1A8*_rs1042597), SNP29 (*CYP2C18*_rs1126545), SNP3 (*CYP2D6*_rs16947), SNP15 (*UGT1A10*_rs1105879), and SNP27 (*CYP2B6*_rs3745274). The synergistic cluster contained 14 unique genes including *CYP2D6, UGT1A8, UGT1A10, CYP2E1, CYP3A7, CYP3A5, UGT2B7, ABCC2, CYP2B6, CYP2C18, SLCO1B1, AKR1C3, UGT1A7,* and *SLC22A1* (Table 3). These 14 genes were considered as the main source of further in silico analyses as follows: signaling pathways in PPIs, GMIs, PCIs, and PDIs.

**Table 3:** The variants (SNPs) numbers and their references utilized in MDR analysis and ViSEN

SNP NO	RS ID	Gene
SNP1	rs2273697	*ABCC2*
SNP2	rs1135840	*CYP2D6*
SNP3	rs16947	*CYP2D6*
SNP4	rs1138272	*GSTP1*
SNP5	rs1695	*GSTP1*
SNP6	rs1042008	*SULT1A1*
SNP7	rs1042028	*SULT1A1*
SNP8	rs1801030	*SULT1A1*
SNP9	rs6759892	*UGT1A6*
SNP10	rs2070959	*UGT1A8*
SNP11	rs17868323	*UGT1A10*
SNP12	rs11692021	*UGT1A10*
SNP13	rs1105879	*UGT1A10*
SNP14	rs72551330	*UGT1A9*
SNP15	rs1105879	*UGT1A10*
SNP16	rs11568681	*ABCC4*
SNP17	rs145014075	*CYP2A6*
SNP18	rs1135840	*CYP2D6*
SNP19	rs2515641	*CYP2E1*
SNP20	rs1799853	*CYP2C9*
SNP21	rs2257401	*CYP3A7*
SNP22	rs776746	*CYP3A5*
SNP23	rs28365063	*UGT2B7*
SNP24	rs7439366	*UGT2B7*
SNP25	rs7438284	*UGT2B7*
SNP26	rs3740066	*ABCC2*
SNP27	rs3745274	*CYP2B6*
SNP28	rs3211371	*CYP2B6*
SNP29	rs1126545	*CYP2C18*
SNP30	rs2270860	*SLC22A7*
SNP31	rs11045819	*SLCO1B1*
SNP32	rs2306283	*SLCO1B1*
SNP33	rs2306168	*SLCO2B1*
SNP34	rs7439366	*UGT2B7*
SNP35	rs12529	*AKR1C3*
SNP36	rs324420	*FAAH*
SNP37	rs1799983	*NOS3*
SNP38	rs3842787	*PTGS1*
SNP39	rs1751034	*ABCC4*
SNP40	rs11572078	*CYP2C8*
SNP41	rs2306283	*SLCO1B1*
SNP42	rs4149117	*SLCO1B3*
SNP43	rs60140950	*SLCO1B3*
SNP44	rs4513095	*CES1*
SNP45	rs17863778	*UGT1A7*
SNP46	rs138417770	*CYP2D6*
SNP47	rs717620	*ABCC2*
SNP48	rs8187710	*ABCC2*
SNP49	rs2279343	*CYP2B6*
SNP50	rs12208357	*SLC22A1*
SNP51	rs628031	*SLC22A1*
SNP52	rs683369	*SLC22A1*
SNP53	rs1042597	*UGT1A8*
SNP54	rs4244285	*CYP2C19*

The data of this table represent the details related to the SNPs utilized in the ViSEN tool. No means number and SNP refers to Single Nucleotide Polymorphism

**Fig. 3:**
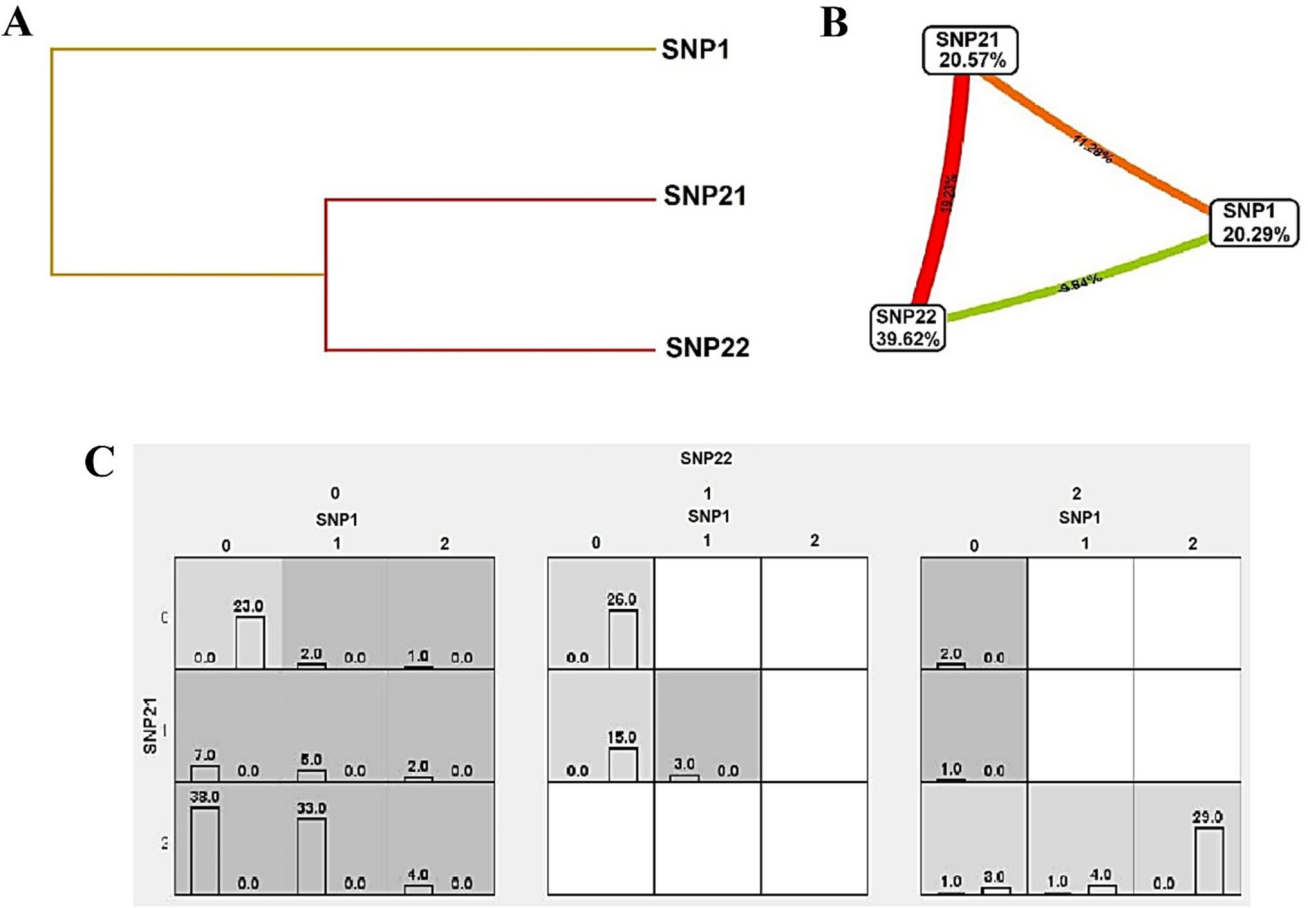
The best model of three SNPs interactions are represented by **A** dendrogram, **B** Fruchterman-Reingold model, and **C** graphical model based on carriers’ genotypes status including Dominant Homozygotes (Major/Major alleles = 0), Heterozygotes (Major/minor alleles = 1), and Recessive homozygotes (minor/minor alleles = 2). Notably, grey squares reflect most significant synergistic interactions. In this figure, part A demonstrates the most significant synergistic relationship among three SNPs out of 54 primary SNPs; in part B, we can observe their relationships in numbers which in turn, magnifies the top-scored relationship inside these three SNPs, and finally, part C uncovers their associations in specific allelic-level details

The best model analyzed and subsequently indicated by MDR was an entropic relationship between SNP1 (*ABCC2*_rs2273697), SNP21 (*CYP3A7*_rs2257401), and SNP22 (*CYP3A5*_ rs776746). Cross Validation (CV) of SNP1, SNP21, and SNP22 included Training Balanced Accuracy: 0.9906, Training Χ^2^: 173.3261 (*p* < 0.0001), Training Sensitivity: 0.9811, Training Accuracy: 0.9906, Training Specificity: 1.0, Training Kappa: 0.9811, Training Precision: 1.0, and Training F-Measure: 0.9905. Dendrogram and Fruchterman-Reingold models indicated that there is a strong synergy among rs2257401 and rs776746 (19.23%); also, we found another synergism that was revealed among rs2273697 and rs2257401 (11.28%). According to the overall balanced accuracy, there are other important synergistic relationships among other SNPs; for example, among SNP6 (*SULT1A1*_rs1042008), SNP21 (*CYP3A7*_rs2257401), and SNP22 (*CYP3A5*_rs776746). Furthermore, a Graphical Model uncovered the genotypic relevance among these three SNPs (Fig. 3). Notably, rs2273697 and rs2257401 are both nsSNV and rs776746 is a splicing variant.

### Three-Dimensional Gene–Gene Interactions (GGIs)

**Fig. 4:**
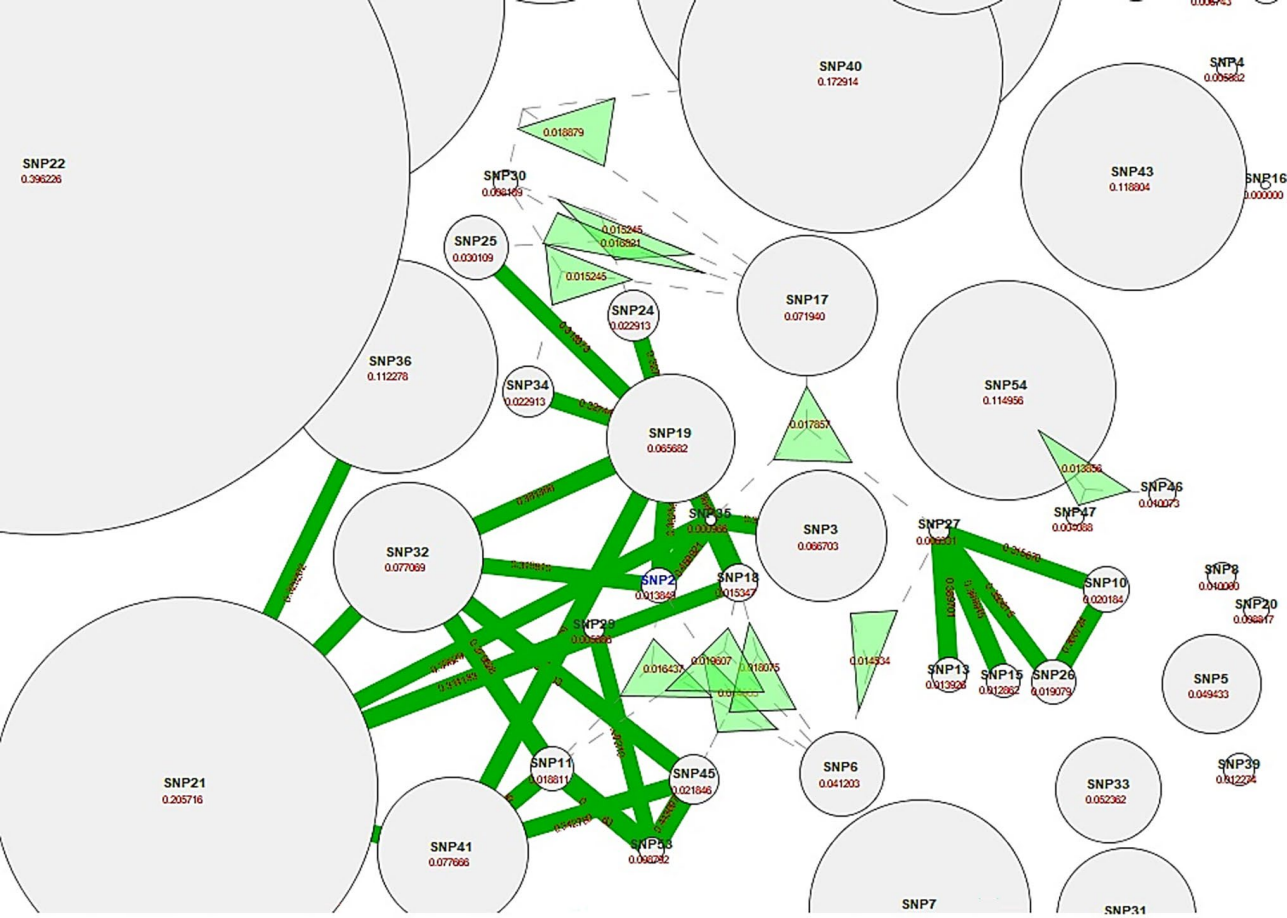
The 2-way and 3-way gene–gene interactions of 54 variants visualized by ViSEN. The triangles specify the three-ways interactions among the SNPs. In this image, the most effective SNPs are the bigger circles and two-way interactions can be seen by dark green lines; however, the green triangles represent the three-way interactions which highlight the utmost impacts of related SNPs (each SNP with a three-way interaction links to one angle of a bright green triangle)

ViSEN software analyzes and visualizes non-linear interactions between discrete characteristics, such as SNPs, that predict a discrete outcome, like a case–control condition used in our study. The ViSEN program quantifies both pairwise and 3-way epistatic interactions using single information-gain measures. It visualizes three orders of effects, that is, main effects, pairwise and 3-way interactions, in one network at the same time. In Fig. 4 the circular nodes stand for qualities, the solid-line edges mean pairwise connections, and the triangles are 3-way connections. The geometric area forms and the breadth of their edges show their power (Hu et al. 2011, 2013b, 2013c). To reach a deeper insight into the SNP-SNP interactions, we utilized ViSEN for all 54 SNPs to find three-way interactions. A network visualized by ViSEN showed the top 2-way interactions threshold of 0.314 and the top 3-way interactions threshold of 0.0319. Amonst them, this 2-way, 3-way interacted network confirmed MDR results and added 3-way interactions for multiple SNPs.Specifically, SNP17 and SNP6 had the highest 3-way interactions (both 2- and 3-way interactions) (Fig. 4) (Supplementary Table 1).

### Protein–Protein Interactions (PPIs)

**Fig. 5:**
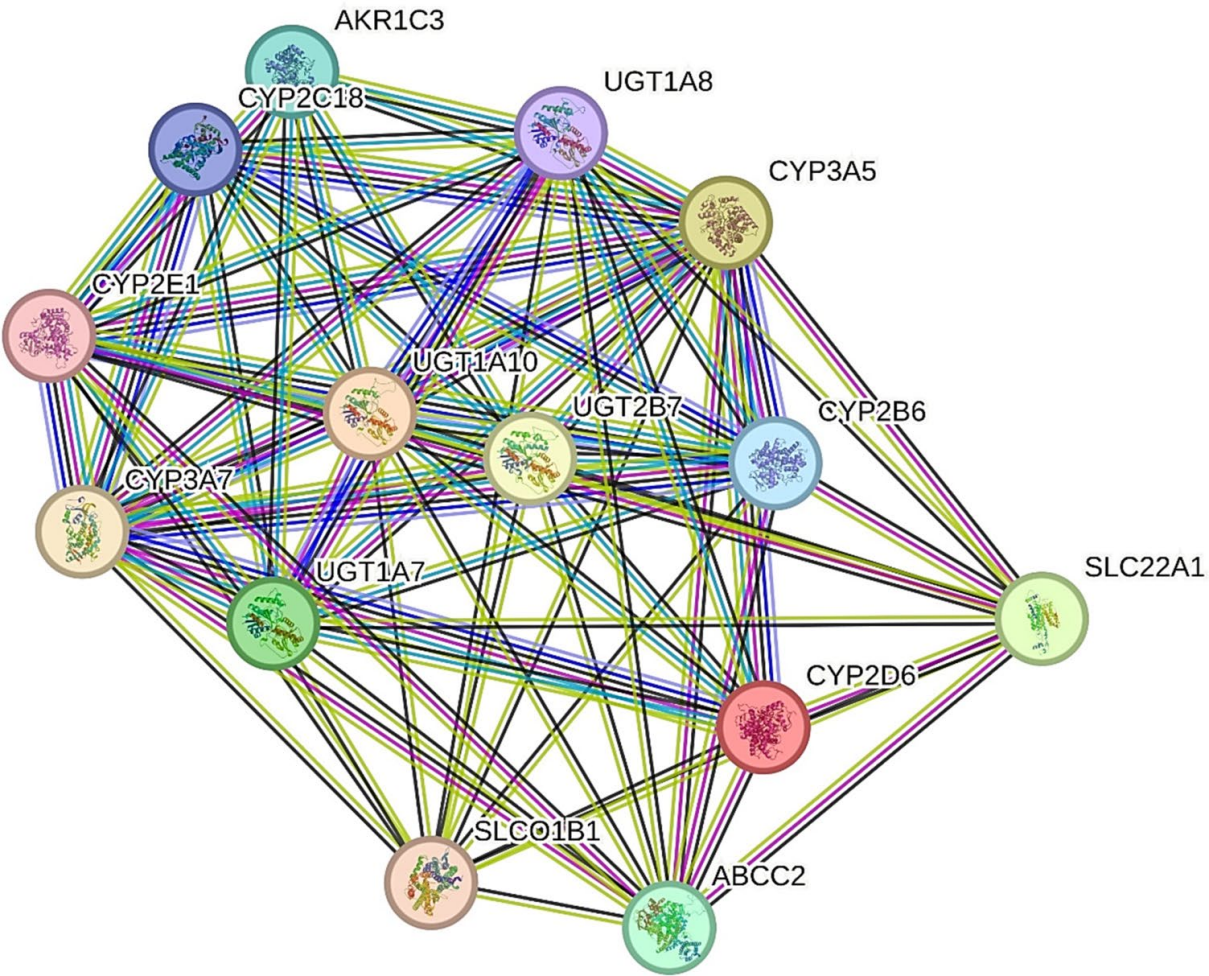
STRING-MODEL of 14 human genes resulted from the 21 synergistic cluster of MDR outputs. This PPI network confirms the physical interactions of synergistic variants as the candidate SNPs of their related genes with each other. Indicating the Proteogenomic potential of final MDR-based genes, this PPI network consists of Nodes and Edges; Every protein produced by a single protein-coding gene locus is represented by a node that indicates proteins (splice isoforms or post-translational modifications are collapsed), and edges show protein–protein associations (associations are meant to be specific and meaningful, i.e., proteins jointly contribute to a shared function; this does not necessarily mean they are physically binding to each other). Additionally, each colored line stands for an evidenced-based, predicted, or other interaction as follows: light Blue refers to interaction from reliable databases and Pink refers to experimentally detected (known interactions), dark green refers to gene neighborhoods, red stands for gene fusions, dark blue shows gene co-occurrences (Predicted interactions), light green reflects text-mining, black signifies the co-expressions, and velvet comes from protein-homology (Other interactions)

To validate the PPIs among 14 candidate genes, the STRING-MODEL of these genes were utilized, and the primary outcome results revealed that all of these genes are connected together according to the strong molecular evidence displayed with a PPI enrichment *p*-value lower than 1.0e-16 (Fig. 5).

### Signaling Pathway Analysis (SPA)

**Fig. 6:**
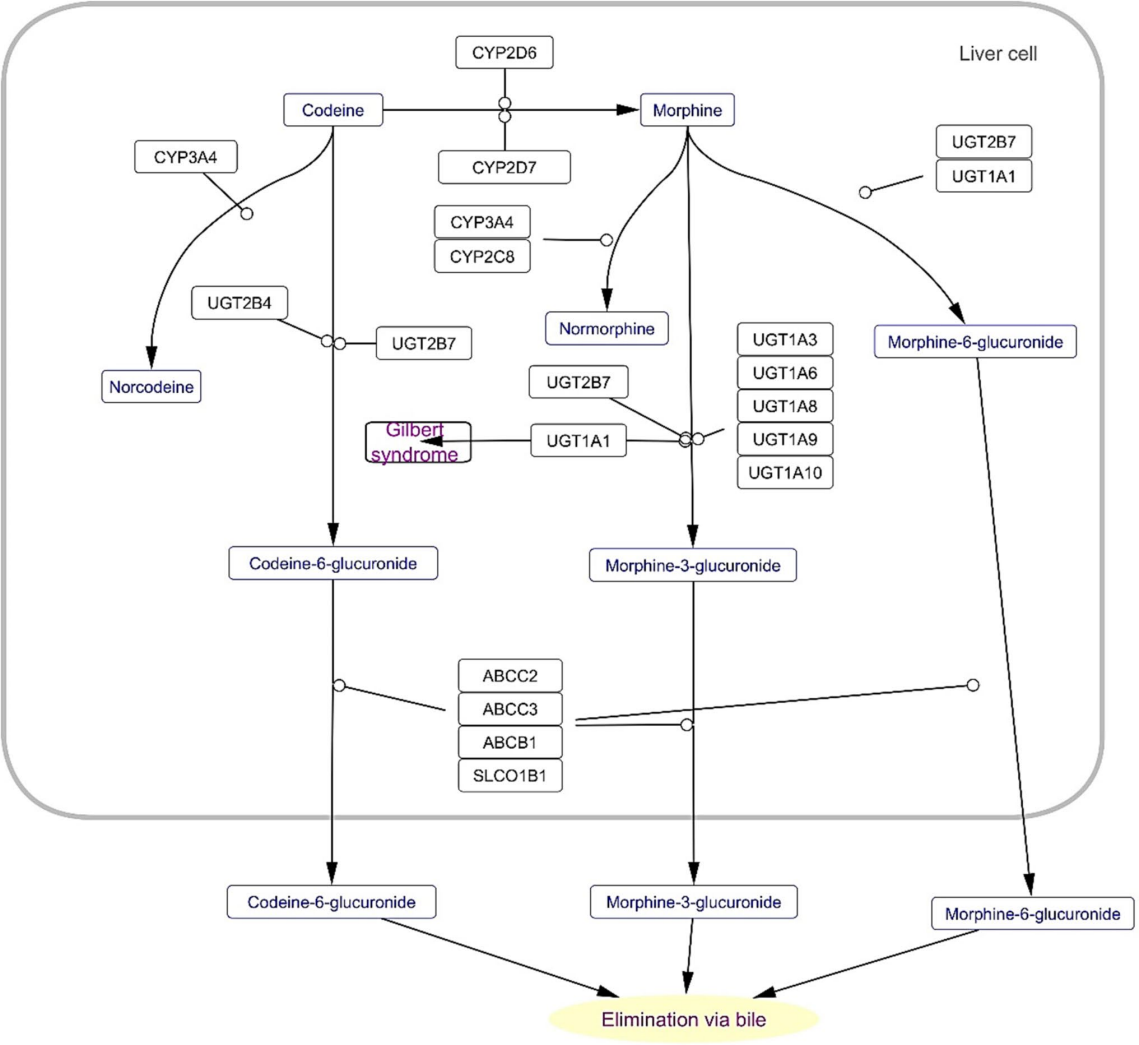
Codeine and morphine Metabolism pathway as the most significant curated signaling pathway with a *p*-value of 3.69e-13 envisaged by Cytoscape. This illustration confirms the major impact of Pain signaling pathways in the PAIma panel

Employing Cytoscape ver. 3.10.1, the most significant and curated signaling pathway containing 14 genes was codeine and morphine Metabolism pathway with a *p*-value of 3.69e-13. Cytoscape also showed that Tamoxifen metabolism pathway was the second significant curated pathway (*p* = 1.66e-12) (Fig. 6).

### Gene-miRNA Interactions (GMIs)

**Fig. 7:**
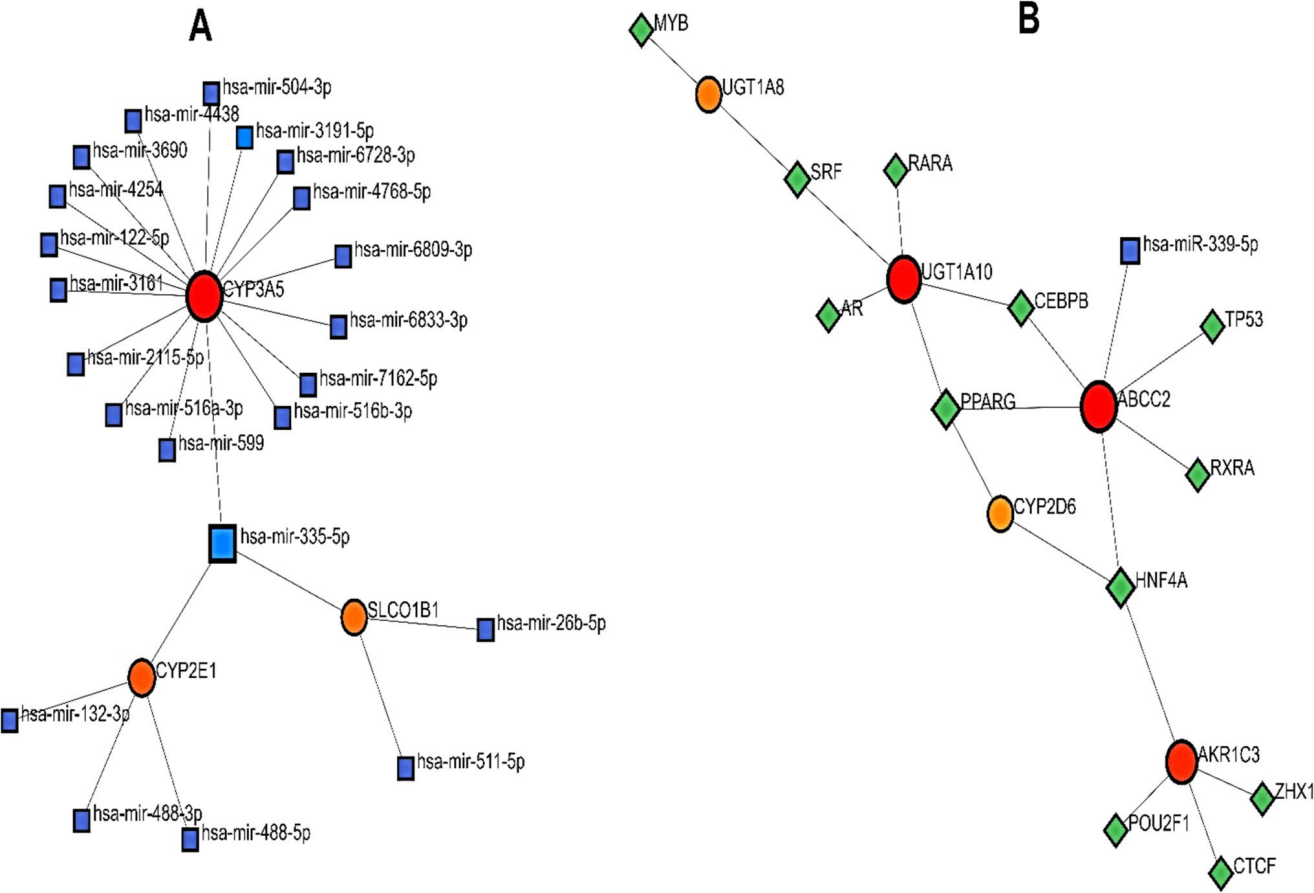
**A** GMIs and **B** TF-miR coregulatory network both in Fruchterman-Reingold models performed via NetworkAnalyst. Both of these shapes reveal the importance of 7 genes among 14 genes extracted from MDR analysis. In fact, these networks emphasize the expression potential of these genes in Transcriptomics

GMIs through NetworkAnalyst with adjusting the GMIs according to the miRTarBase v8.0 revealed the First-Order Network in some sub-networks which among them, *CYP3A5, CYP2E1*, and *SLCO1B1* genes had the most important miRNA connections. GMIs revealed that hsa-miR-355-5p is very important due to its links with all of the aforementioned three genes. The backbone model of GMIs was selected to show the gene-miRNA connections (Fig. 7A). Furthermore, TF-miR Coregulatory Network was applied to this gene list and notable outputs were obtained. The literature validated regulatory interaction data were gathered from the RegNetwork (http://www.regnetworkweb.org/) repository. Some Transcription factors (TFs) showed multiple interactions with the mentioned genes including PPARG which was related to *ABCC2, CYP2D6*, and *UGT1A10*; HNF4A which had associations with *ABCC2*, *CYP2D6*, and *AKR1C3*; and SRF which was connected to the *UGT1A10, UGT1A7*, and *UGT1A8* (Fig. 7B).

### Protein-Drug Interactions (PDIs)

The protein and drug target information, which was utilized by NetworkAnalyst, was obtained from the DrugBank database (Version 5.0). PDIs indicated multiple separate subnetworks. In fact, one example for a separate subnetwork was paliperidone as a FDA-approved drug with more than one gene target (*CYP2D6* and *CYP3A5*) (Figure not shown).

### Protein-Chemical Interactions (PCIs)

**Fig. 8:**
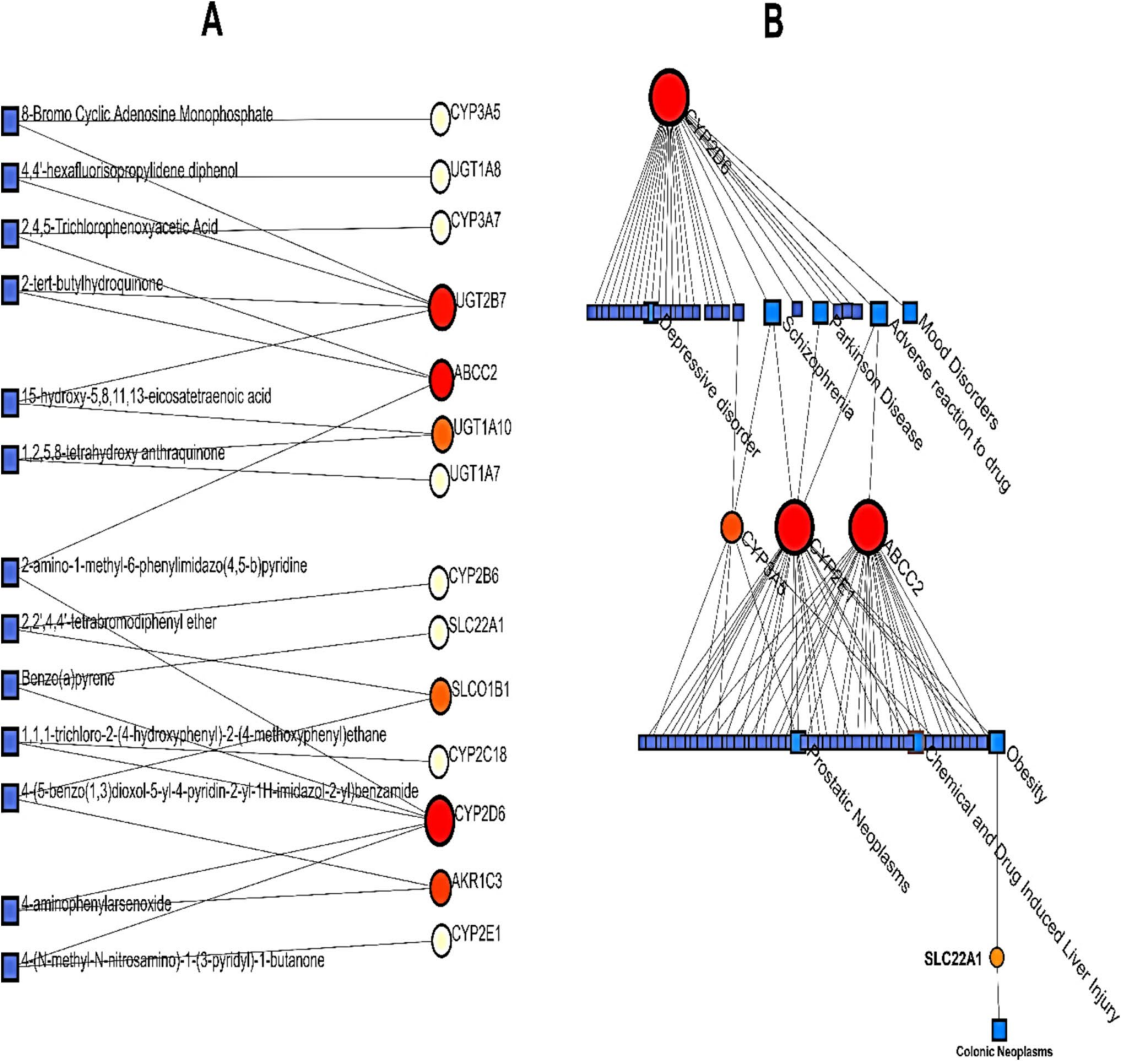
**A** Linear Bi/Tripartite model of PCIs and **B** Sugiyama model of GDAs both assessed and illustrated by NetworkAnalyst. Briefly, these networks indicate the potential involvement of the MDR findings in Phenomics and may be important for personalized medicine treatment requiring future clinical confirmations

The data of PCIs in NetworkAnalyst was on the basis of data from the Comparative Toxicogenomics Database (CTD) and the findings from PCIs showed that the most interactive gene is *CYP2D6* and the highest interaction degree is 2 for chemicals. In a Linear Bi/Tripartite model of PCIs, some chemicals might be candidates for future drug discovery such as 4-(N-methyl-N-nitrosamino)-1-(3-pyridyl)-1-butanone (linked to *CYP2D6* and *CYP2E1*), 4-aminophenylarsenoxide (associated with *CYP2D6* and *ARK1C*), 1,1,1-trichloro-2-(4-hydroxyphenyl)-2-(4-methoxyphenyl)ethane (interacted with *CYP2D6* and *CYP2C18*)*,* Benzo(a)pyrene (connected to the *CYP2D6* and *SLC22A1*), and finally, 2-amino-1-methyl-6-phenylimidazo(4,5-b)pyridine (had relationships with *CYP2D6* and *ABCC2*). The other chemicals can be found in Fig. 8A.

### Gene-Disease Associations (GDAs)

GDAs were investigated to find the most important genes involving various diseases by NetworkAnalyst which utilizes the literature curated GDA information from the DisGeNET database. While the Sugiyama model indicated that *CYP2D6* had the highest degree of betweenness; both *CYP2E1* and *CYP3A5* trigger common diseases with *CYP2D6*. For comprehension purposes it is known that adverse drug reactions (ADRs), Drug Allergy, chemical and drug-induced liver injury, Parkinson’s disease, Schizophrenia, and Mood disorders are some of the diseases in associations with *CYP2D6*, *CYP2E1*, and *CYP3A5* genes (Fig. 8B).

In summary, we believe that our approach described herein, involving pretesting investigations focusing on a putative major signaling pathway category (PAIma including 21 curated pathway) led to raw big data annotation (55,590 variant annotations) which in our case was further filtered multiple times to enable required refinement (54 functional nsSNVs and regulatory variants remained). These refined variants were related to the initial 128 pharmacogenes and WES tests (VCFs) of 100 Western Iranians analyzed by this primary gene list or gene panel through Ubuntu 22.04.2. To our knowledge, this is the first study to utilize a clean life-time non-psychoactive using cohort to apply multiple in deep silico pharmacogenomic layers to promote pharmacogenomics guidelines, future NGS analyzers, and company-based sites such as Centogene (https://www.centogene.com/), Fulgent Genetics (https://www.fulgentgenetics.com/), DisGeNET (https://www.disgenet.org/), CeGat (https://cegat.com/), Blueprint Genetics (https://blueprintgenetics.com/), Prevention Genetics (https://www.preventiongenetics.com/), Asper Biogene (https://www.asperbio.com/), Invitae (https://www.invitae.com/), etc. In essence we found a new gene–gene interaction strategy designed for MDR analysis (controls vs. pseudo-controls) on the final 54 variants (SNPs) which remained following subsequent analyses. Importantly, MDR results showed a synergistic cluster containing 21 SNPs and 14 related protein-coding genes.

**Fig. 9:**
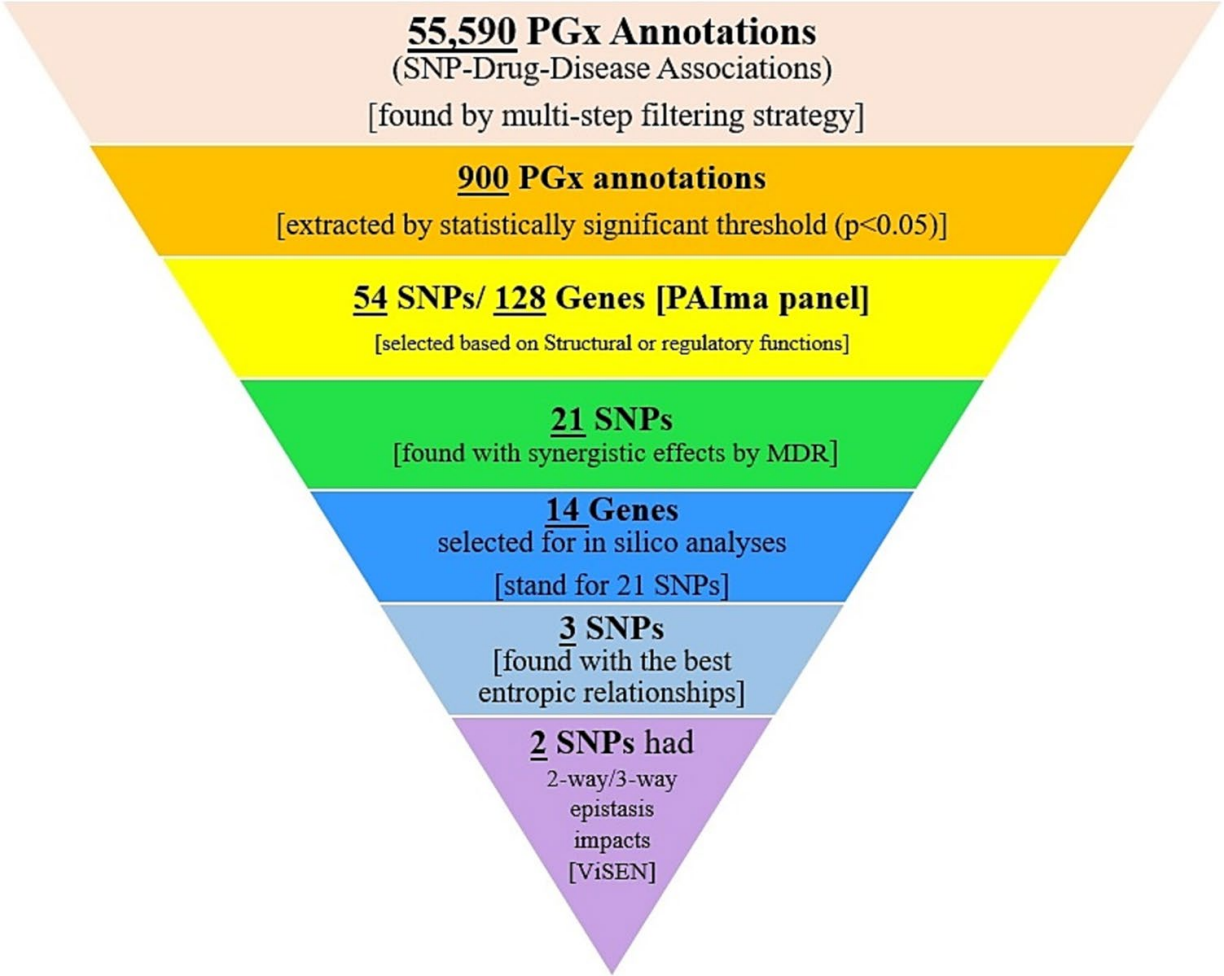
Summary of results in an inverted triangle-divided diagram highlighting the main finding(s) in each fundamental step of this study. Obviously, we introduced 2 or 3 SNPs with highly potential impacts on PAIma panel from a Big Data inclusion (55,590 variant annotations)

Briefly, for readership comprehension, because of the complexity of these heuristic results, we illustrated a high level diagram indicating our final finding(s) in each main step of this investigation from 55,590 PGx annotations (raw data) to MDR and ViSEN results (3 and 2 SNPs, respectively) (Fig. 9).

## Discussion

For the current investigation we performed multiple analyses based on the filtered-based findings of our newly introduced PAIma panel (based on WES results) and novel strategy for MDR analyses. Additionally, we performed multiple in silico analyses that included 3-way GGIs, PPIs, SPA, GMIs, PDIs, PCIs, and GDAs. MDR analyses revealed a synergistic cluster containing 21 SNPs related to 14 genes. In addition, ViSEN indicated rs145014075 (*CYP2A6*) and rs1042008 (*SULT1A1*) having the highest 3-way interactions. GMIs revealed that hsa-miR-355-5p is very important. Interestingly, TF-miR coregulatory network analysis uncovered the metabolizing *CYP2D6* gene as being highly impactful. PDIs found paliperidone as the high-interacted FDA-approved drug associated with *CYP2D6* and *CYP3A5*. PCIs revealed that CYP2D6 had the most relationships with chemicals. Lastly, GDAs highlighted that *CYP2D6* had the highest betweenness. Also, GDAs showed that *CYP2E1* and *CYP3A5* trigger common diseases with *CYP2D6* such as ADRs, Drug Allergy, Chemical and drug-induced liver injury, Parkinson’s disease, Schizophrenia, and Mood disorders. Furthermore, our results revealed fewer but higher potential actionable SNPs (compared to 54 SNPs) for drug prescribing of PAIma pathways among Iranians by genotyping an array of 10 SNPs including rs1135840, rs16947, rs1135840, rs138417770 (*CYP2D6*), rs776746 (*CYP3A5*), rs145014075 (*CYP2A6*), rs2515641 (*CYP2E1*), rs11045819, rs2306283 (*SLCO1B1*), and rs1042008 (*SULT1A1*).

As mentioned earlier, our results related to: *CYP2D6, CYP3A5, CYP3A7, CYP2A6, ABCC2,* and *SULT1A1* indicated important roles in pain, inflammation, and immunity processes. There are remarkable reports in the literature highlighting the associations of pharmacokinetics and pharmacodynamics of these genes involving pain management among healthy people (Ohno et al. 2008; Novalbos et al. 2010; Lohela et al. 2021). However, a few studies investigated the relationships of genetic polymorphisms of these genes with pain management. For instance, a recent systematic review reported by Zondeh et al. conducted on 25 papers (out of the 6547 originally identified publications) to study related drug–gene relationships which were distinguished for the drug safety yielded some remarkable results. These authors found important medication–gene interactions in pain management including ibuprofen with *CYP2C9*; celecoxib with *CYP2C9*; piroxicam with *CYP2C8* and *CYP2C9*; diclofenac with *CYP2C9*, *CYP2C8*, *UGT2B7*, and *ABCC2*; meloxicam with *CYP2C9*; aspirin with *SLCO1B1*, *CYP2C9*, and *CHST2*; amitriptyline with *CYP2C19* and *CYP2D6*; imipramine with *CYP2C19*; nortriptyline with *CYP2D6*, *CYP2C19*, and *ABCB1*; and lastly, escitalopram with *HTR2C*, *CYP2C19*, and *CYP1A2* (Zobdeh et al. 2022). Interestingly, in a Randomized Clinical Trial by Pickering et al., polymorphisms of 23 receptors and enzymes were investigated, and associations were found with pain alleviation among 47 Caucasian Healthy Volunteers. These investigators described the association of *SULT1A1* SNP (rs224534) with paracetamol anti-nociception (Pickering et al. 2020). All of these studies are consistent with our investigation. In contrast to PGx associations of PAIma panel genes among healthy cohorts, Mejía-Abril et al. involved 85 healthy individuals in 3 clinical trials and informed that there were no associations between the genetic polymorphisms of *CYP2D6, CYP3A5,* *ABCB1, CYP3A4,* *ABCC2, UGT1A1, SLCO1B1, CYP1A2, CYP2A6*, *CYP2C8, CYP2B6, CYP2C19, CYP2C9* and *SLC22A1* with the adverse impacts of dexketoprofen as a NSAID (Mejía-Abril et al. 2021).

We are cognizant that using powerful opioids and other psychoactive drugs even at an addictive rate will not alter DNA antecedents or the presence of such polymorphisms but will indeed affect miRNA transcription epigenetically for at least f2 generations (Hamilton and Nestler 2019). Other reports have acknowledged the significance of inter-individual genetic varieties in buprenorphine metabolism revealing variable treatment feedback, these genetic variations are the cause of treatment failures for some patients and making them highly vulnerable for relapse. Accordingly, Ettienne et al. made a strong case for clinical pharmacogenomics studies as revealed herein, to profoundly affect opioid prescribing based on one’s inherited genetic variations and their subsequent drug response (Ettienne et al. 2017). Specifically, in their study, PGx testing demonstrated that African-American patients presented a cytochrome P450 3A4 (CYP3A4) ultra-rapid metabolizer phenotype necessitating an elevated than suggested daily dosage of buprenorphine for suitable OUD administration (32 mg). By comparing the patient’s relapse rate to usual dosing, the pharmacogenetic-linked dose recommendation showed a significant reduction in relapses, which is an important recovery outcome.

It is indeed noteworthy that in the current study, albeit a rather modest population requiring much larger samples and a variety of ethnic groups, prior to any generalizations that could be made, the results of this first ever approach and subsequent map of 14 genes seems quite parsimonious. Certainly, confirmation of these results will open a new pathway in recovery science to assist in both prescribing methadone and buprenorphine to reduce harm in an addictive population and or even to treat ongoing pain (Lee et al. 2024). The take home message herein is to highlight the effect of PGx testing on OUD managing results.

Finally, one important feature of these findings related to pharmacogenomics gene–gene interactions, can be potentially useful in the future as a more accurate way clinicians could prescribe opioid medications for pain and or harm reduction (Adams et al. 2023; Suarez et al. 2023; Johnson et al. 2024). This may involve, prior to prescribing these powerful addictive pharmaceuticals, especially to a pre-addictive genetic or epigenetic population, genotyping variants as denoted herein as an initial panel (McLellan et al. 2022; Blum et al. 2024). However, we believe that both time and cost-savings might be assisted via molecular techniques such as Multiplex-PCR and Real-time PCR methods before utilizing the WES test (which takes weeks and months for preparations and interpretations of an individual’s drug susceptibilities). We believe that these results are encouraging and may provide a gene–gene map having heuristic value to help reduce the global public health opioid crisis (Blum et al. 2020; Bakkali et al. 2023; McKenzie-Brown et al. 2023; Muir et al. 2023; De Aquino et al. 2024).

## Limitations

While we are cognizant that one hundred participants, albeit significant findings from our study herein, a larger cohort employing this PGx approach should help refute or confirm our potentially interesting results. Based on this investigation we are proposing the possible potential flexibility of pharmacogenomics to enable the prescribing clinician to utilize these findings related for example to develop a panel of 14 annotated genes as a guide to assist in the prescribing of powerful opioids for not only pain management but for recovery maintenance as well.

Following confirmation of our results, albeit an Iranian population, this type of work could serve as a model going forward. While we tried our best to control the power of the study, it is highly notable that future studies performing MDR, consider the MAFs. The most interesting finding of this investigation promotes a personalized medicine approach that links our WES results and known prescribing FDA-approved drugs involved in PAIma pathways by focusing on the most important variants form 55,590 variants annotations to the 21 actionable alleles found in a synergistic cluster of GGIs. Simply, the novelty of our primary findings coupling both WES and PAIma with the MDR analyses may lead to the futuristic development of real personalized medicine to help enable higher precision levels in prescribing the related FDA-approved analgesics like opioid type drugs awaiting other countries to also test populations similarly. As such we are cognizant that ethnicity (race) may vary accordingly and our panel of 14 annotated genes may differ from country to country.

## Conclusion

The goal of the current study was to create an updated and comprehensive PGx gene panel (PAIma) focusing on pain based on GGI using MDR and ViSEN. Our study herein considered pain pathways and its related variants, genes, and drugs (methadone, morphine, nicotine, and amphetamine). Moreover, in an attempt to capture novel personalized PGX medicine, we hereby investigated the susceptibility and predisposition potential of healthy people to pain and psychiatric medications by displaying their associated PGx-related variants. It is highly recommended that a translation of this work to clinical utility is using the PAIma panel (128 Pharmacogenes) in NGS analyses, targeted therapy, Prescribing the right pharmaceuticals with effective doses including analgesics, anti-psychotics, NSAIDs, Chemotherapeutic agents, Anti-viral medications, and transplantation- anti -rejection agents. Undertaking the analyses of our strategy might prove worthwhile to the entire scientific community to reduce the well-described global public health opioid misuse crisis. Finally, it is quite possible that adoption of our technique could provide a higher level of personalized medicine and precision opioid type therapy and as such maybe the new norm.

## Supplementary Information

Below is the link to the electronic supplementary material.

Supplementary file1 (DOCX 15 KB)

## Data Availability

The datasets obtained and studied during this research are accessible in the following repositories: dbSNP [https://www.ncbi.nlm.nih.gov/snp/https://www.ncbi.nlm.nih.gov/snp/] and clinVAR [https://www.ncbi.nlm.nih.gov/clinvar/https://www.ncbi.nlm.nih.gov/clinvar/]. Data will be available on demand to the corresponding author Dr. Majid Motovali-bashi via mbashi@sci.ui.ac.ir.

## References

[CR1] Adams JW, Duprey M, Khan S, Cance J, Rice DP, Bobashev G (2023) Examining buprenorphine diversion through a harm reduction lens: an agent-based modeling study. Harm Reduct J 20(1):150 37848945 10.1186/s12954-023-00888-6 PMC10580611

[CR2] Bakkali N, Ott L, Triquet C, Cottencin O, Grynberg D (2023) Learning from others’ experience: social fear conditioning deficits in patients with severe alcohol use disorder. Alcohol Clin Exp Res 47(8):1603–1613 10.1111/acer.15129 37573573

[CR3] Blum K, Lott L, Baron D, Smith DE, Badgaiyan RD, Gold MS (2020) Improving naltrexone compliance and outcomes with putative pro-dopamine regulator KB220, compared to treatment as usual. J Syst Integr Neurosci. 10.15761/JSIN.1000229 32934823 10.15761/JSIN.1000229 PMC7489288

[CR4] Blum K, Bowirrat A, Thanos PK, Hanna C, Khalsa J, Baron D, Elman I, Badgaiyan RD, Dennen C, Braverman ER (2024) Evidence based clinical analytics supporting the genetic addiction risk severity (GARS) assessment to early identify probands in preaddiction. EC Psychol Psychiatr 13(1):1–3 38298272 PMC10825809

[CR5] Consortium, I. M. S. G. (2004) Enhancing linkage analysis of complex disorders: an evaluation of high-density genotyping. Hum Mol Genet 13(17):1943–1949 15238506 10.1093/hmg/ddh202

[CR6] Culverhouse R, Klein T, Shannon W (2004) Detecting epistatic interactions contributing to quantitative traits. Genet Epidemiol 27(2):141–152 15305330 10.1002/gepi.20006

[CR7] Dai H, Charnigo RJ, Becker ML, Leeder JS, Motsinger-Reif AA (2013) Risk score modeling of multiple gene to gene interactions using aggregated-multifactor dimensionality reduction. BioData Min 6(1):1–16 23294634 10.1186/1756-0381-6-1 PMC3560267

[CR8] De Aquino JP, Sloan ME, Nunes JC, Costa GP, Katz JL, de Oliveira D, Ra J, Tang VM, Petrakis IL (2024) Alcohol use disorder and chronic pain: an overlooked epidemic. Am J Psychiatry 181(5):391–402 38706339 10.1176/appi.ajp.20230886 PMC11521207

[CR9] Ettienne EB, Chapman E, Maneno M, Ofoegbu A, Wilson B, Settles-Reaves B, Clarke M, Dunston G, Rosenblatt K (2017) Pharmacogenomics-guided policy in opioid use disorder (OUD) management: an ethnically-diverse case-based approach. Addict Behav Rep 6:8–14 29450233 10.1016/j.abrep.2017.05.001 PMC5800559

[CR10] Hahn LW, Ritchie MD, Moore JH (2003) Multifactor dimensionality reduction software for detecting gene–gene and gene–environment interactions. Bioinformatics 19(3):376–382 12584123 10.1093/bioinformatics/btf869

[CR11] Hamilton PJ, Nestler EJ (2019) Epigenetics and addiction. Curr Opin Neurobiol 59:128–136 31255844 10.1016/j.conb.2019.05.005 PMC6889055

[CR12] Hoh J, Wille A, Ott J (2001) Trimming, weighting, and grouping SNPs in human case-control association studies. Genome Res 11(12):2115–2119 11731502 10.1101/gr.204001 PMC311222

[CR13] Hu T, Sinnott-Armstrong NA, Kiralis JW, Andrew AS, Karagas MR, Moore JH (2011) Characterizing genetic interactions in human disease association studies using statistical epistasis networks. BMC Bioinform 12(1):1–13 10.1186/1471-2105-12-364 PMC3215301 21910885

[CR14] Hu T, Andrew AS, Karagas MR, Moore JH (2013a) Statistical epistasis networks reduce the computational complexity of searching three-locus genetic models. Biocomputing 2013. World Scientific, Singapore, pp 397–408 PMC3587773 23424144

[CR15] Hu T, Chen Y, Kiralis JW, Collins RL, Wejse C, Sirugo G, Williams SM, Moore JH (2013b) An information-gain approach to detecting three-way epistatic interactions in genetic association studies. J Am Med Inform Assoc 20(4):630–636 23396514 10.1136/amiajnl-2012-001525 PMC3721169

[CR16] Hu T, Chen Y, Kiralis JW, Moore JH (2013c) Vi SEN: methodology and software for visualization of statistical epistasis networks. Genet Epidemiol 37(3):283–285 23468157 10.1002/gepi.21718 PMC3758133

[CR17] Johnson B, Monwell B, Capusan AJ (2024) Long-acting injectable depot buprenorphine from a harm reduction perspective in patients with ongoing substance use and multiple psychiatric comorbidities: a qualitative interview study. Harm Reduct J 21(1):68 38528531 10.1186/s12954-024-00984-1 PMC10964574

[CR18] Lee YK, Gold MS, Fuehrlein BS (2022) Looking beyond the opioid receptor: a desperate need for new treatments for opioid use disorder. J Neurol Sci 432:120094 34933249 10.1016/j.jns.2021.120094

[CR19] Lee YK, Gold MS, Blum K, Thanos PK, Hanna C, Fuehrlein BS (2024) Opioid use disorder: current trends and potential treatments. Front Public Health 11:1274719 38332941 10.3389/fpubh.2023.1274719 PMC10850316

[CR20] Lohela TJ, Poikola S, Neuvonen M, Niemi M, Backman JT, Olkkola KT, Lilius TO (2021) Rifampin reduces the plasma concentrations of oral and intravenous hydromorphone in healthy volunteers. Anesth Analg 133(2):423–434 33177323 10.1213/ANE.0000000000005229 PMC8257471

[CR21] Lord PG, Papoian T (2004) Genomics and drug toxicity. Am Assoc Adv Sci 306:575–575 10.1126/science.1105854 15498974

[CR22] McCarty CA, Wilke RA, Giampietro PF, Wesbrook SD, Caldwell MD (2005) Marshfield Clinic Personalized Medicine Research Project (PMRP): design, methods and recruitment for a large population-based biobank. Per Med. 10.1517/17410541.2.1.49 29793241 10.1517/17410541.2.1.49

[CR23] McKenzie-Brown AM, Boorman DW, Ibanez KR, Agwu E, Singh V (2023) Low-dose naltrexone (LDN) for chronic pain at a single institution: a case series. J Pain Res. 10.2147/JPR.S389957 37337611 10.2147/JPR.S389957 PMC10276990

[CR24] McLellan AT, Koob GF, Volkow ND (2022) Preaddiction—a missing concept for treating substance use disorders. JAMA Psychiatr 79(8):749–751 10.1001/jamapsychiatry.2022.1652 35793096

[CR25] Mejía-Abril G, Zubiaur P, Navares-Gómez M, Villapalos-García G, Román M, Ochoa D, Abad-Santos F (2021) Dexketoprofen pharmacokinetics is not significantly altered by genetic polymorphism. Front Pharmacol 12:660639 33995083 10.3389/fphar.2021.660639 PMC8117330

[CR26] Moore JH (2003) The ubiquitous nature of epistasis in determining susceptibility to common human diseases. Hum Hered 56(1–3):73–82 14614241 10.1159/000073735

[CR27] Moore JH, Gilbert JC, Tsai C-T, Chiang F-T, Holden T, Barney N, White BC (2006) A flexible computational framework for detecting, characterizing, and interpreting statistical patterns of epistasis in genetic studies of human disease susceptibility. J Theor Biol 241(2):252–261 16457852 10.1016/j.jtbi.2005.11.036

[CR28] Motsinger AA, Ritchie MD (2006) Multifactor dimensionality reduction: an analysis strategy for modelling and detecting gene-gene interactions in human genetics and pharmacogenomics studies. Hum Genomics 2:1–11 10.1186/1479-7364-2-5-318 PMC3500181 16595076

[CR29] Muir WM, Lo CL, Bell RL, Zhou FC (2023) Multi-animal-model study reveals mutations in neural plasticity and nociception genes linked to excessive alcohol drinking. Alcohol Clin Exp Res 47(8):1478–1493 10.1111/acer.15131 PMC10728351 37336636

[CR30] Novalbos J, López-Rodríguez R, Román M, Gallego-Sandín S, Ochoa D, Abad-Santos F (2010) Effects of CYP2D6 genotype on the pharmacokinetics, pharmacodynamics, and safety of risperidone in healthy volunteers. J Clin Psychopharmacol 30(5):504–511 20814331 10.1097/JCP.0b013e3181ee84c7

[CR31] Oesterle TS, Thusius NJ, Rummans TA, Gold MS (2019) Medication-assisted treatment for opioid-use disorder. Mayo Clin Proc. 10.1016/j.mayocp.2019.03.029 31543255 10.1016/j.mayocp.2019.03.029

[CR32] Ohno T, Nakayama K, Nakade S, Kitagawa J, Ueda S, Miyabe H, Miyata Y, Ohnishi A (2008) Effect of itraconazole on the pharmacokinetics of imidafenacin in healthy subjects. J Clin Pharmacol 48(3):330–334 18218784 10.1177/0091270007310386

[CR33] Pickering G, Creveaux I, Macian N, Pereira B (2020) Paracetamol and pain modulation by TRPV1, UGT2B15, SULT1A1 genotypes: a randomized clinical trial in healthy volunteers. Pain Med 21(4):661–669 30908574 10.1093/pm/pnz037

[CR34] Ritchie MD, Hahn LW, Moore JH (2003) Power of multifactor dimensionality reduction for detecting gene-gene interactions in the presence of genotyping error, missing data, phenocopy, and genetic heterogeneity. Genet Epidemiol 24(2):150–157 12548676 10.1002/gepi.10218

[CR35] Sing CF, Stengård JH, Kardia SL (2004) Dynamic relationships between the genome and exposures to environments as causes of common human diseases. Nutrigenet Nutrigenomics 93:77–91 10.1159/000081252 15496802

[CR36] Suarez E, Bartholomew TS, Plesons M, Ciraldo K, Ostrer L, Serota DP, Chueng TA, Frederick M, Onugha J, Tookes HE (2023) Adaptation of the tele-harm reduction intervention to promote initiation and retention in buprenorphine treatment among people who inject drugs: a retrospective cohort study. Ann Med 55(1):733–743 36856571 10.1080/07853890.2023.2182908 PMC9980015

[CR37] Thornton-Wells TA, Moore JH, Haines JL (2004) Genetics, statistics and human disease: analytical retooling for complexity. Trends Genet 20(12):640–647 15522460 10.1016/j.tig.2004.09.007

[CR38] Wang K, Li M, Hakonarson H (2010) ANNOVAR: functional annotation of genetic variants from high-throughput sequencing data. Nucleic Acids Res 38(16):e164–e164 20601685 10.1093/nar/gkq603 PMC2938201

[CR39] Wilke RA, Carrillo MW, Ritchie MD (2005a) Pacific Symposium on biocomputing–computational approaches for pharmacogenomics. Pharmacogenomics. 10.1517/14622416.6.2.111 15882130 10.1517/14622416.6.2.111

[CR40] Wilke RA, Musana AK, Weber WW (2005b) Cytochrome P450 gene-based drug prescribing and factors impacting translation into routine clinical practice. Per Med. 10.2217/17410541.2.3.213 29793264 10.2217/17410541.2.3.213

[CR41] Wilke RA, Reif DM, Moore JH (2005c) Combinatorial pharmacogenetics. Nat Rev Drug Discov 4(11):911–918 16264434 10.1038/nrd1874

[CR42] Zhou G, Soufan O, Ewald J, Hancock RE, Basu N, Xia J (2019) NetworkAnalyst 3.0: a visual analytics platform for comprehensive gene expression profiling and meta-analysis. Nucleic Acids Res 47(W1):W234–W241 30931480 10.1093/nar/gkz240 PMC6602507

[CR43] Zobdeh F, Eremenko II, Akan MA, Tarasov VV, Chubarev VN, Schiöth HB, Mwinyi J (2022) Pharmacogenetics and pain treatment with a focus on non-steroidal anti-inflammatory drugs (NSAIDs) and antidepressants: a systematic review. Pharmaceutics 14(6):1190 35745763 10.3390/pharmaceutics14061190 PMC9228102

