# Supplementary material for: Synergistic Epistasis and Systems Biology Approaches to Uncover a Pharmacogenomic Map Linked to Pain, Anti-Inflammatory and Immunomodulating Agents (PAIma) in a Healthy Cohort
Source: Cell Mol Neurobiol. 2024 Nov 6;44:74. doi: 10.1007/s10571-024-01504-2 (PMC11541314; doi:10.1007/s10571-024-01504-2)
Supplement: Supplementary file 1 — Supplementary file1 (DOCX 15 KB) [file 10571_2024_1504_MOESM1_ESM.docx]

Supplementary Table 1. All 3-way interactions of SNP17 and SNP6 according to the figure 4.

| **Interaction** | **SNP 17 or SNP6** | **Linked SNPs (gene-rsID)** | **Gene** | **RsID** |
| --- | --- | --- | --- | --- |
| 1 | SNP17 | SNP24 | *UGT2B7* | rs7439366 |
|  |  | SNP30 | *SLC22A7* | rs2270860 |
| 2 | SNP17 | SNP25 | *UGT2B7* | rs7438284 |
|  |  | SNP30 | *SLC22A7* | rs2270860 |
| 3 | SNP17 | SNP25 | *UGT2B7* | rs7438284 |
|  |  | SNP34 | *UGT2B7* | rs7439366 |
| 4 | SNP17 | SNP30 | *SLC22A7* | rs2270860 |
|  |  | SNP40 | *CYP2C8* | rs11572078 |
| 5 | SNP17 | SNP2 | *CYP2D6* | rs1135840 |
|  |  | SNP27 | *CYP2B6* | rs3745274 |
| 6 | SNP6 | SNP3 | *CYP2D6* | rs16947 |
|  |  | SNP27 | *CYP2B6* | rs3745274 |
| 7 | SNP6 | SNP18 | *CYP2D6* | rs1135840 |
|  |  | SNP45 | *UGT1A7* | rs17863778 |
| 8 | SNP6 | SNP11 | *UGT1A10* | rs17868323 |
|  |  | SNP18 | *CYP2D6* | rs1135840 |
| 9 | SNP6 | SNP2 | *CYP2D6* | rs1135840 |
|  |  | SNP45 | *UGT1A7* | rs17863778 |
| 10 | SNP6 | SNP2 | *CYP2D6* | rs1135840 |
|  |  | SNP11 | *UGT1A10* | rs17868323 |

This table describes the potential 3-way interactions of SNP17 (*CYP2A6*_rs145014075) and SNP6 (*SULT1A1*_rs1042008) with other SNPs based on ViSEN results. Rs ID refers to Reference sequence ID obtained from dbSNP [NCBI].
